# An altered cytotoxic program of CD8^+^ T-cells in HIV-infected patients despite HAART-induced viral suppression

**DOI:** 10.1371/journal.pone.0210540

**Published:** 2019-01-09

**Authors:** Federico Perdomo-Celis, Paula A. Velilla, Natalia A. Taborda, María Teresa Rugeles

**Affiliations:** 1 Grupo Inmunovirología, Facultad de Medicina, Universidad de Antioquia, UdeA, Medellín, Colombia; 2 Grupo de Investigaciones Biomédicas Uniremington, Programa de Medicina, Facultad de Ciencias de la Salud, Corporación Universitaria Remington, Medellín, Colombia; National Institute of Allergy and Infectious Diseases, UNITED STATES

## Abstract

Despite the suppression of viral replication induced by the highly active anti-retroviral therapy (HAART), an increased immune activation and inflammatory state persists in HIV-infected patients, contributing to lower treatment response and immune reconstitution, and development of non-AIDS conditions. The chronic activation and inflammation affect the functionality and differentiation of CD8^+^ T-cells, particularly reducing their cytotoxic capacity, which is critical in the control of HIV replication. Although previous studies have shown that HAART induce a partial immune reconstitution, its effect on CD8^+^ T-cells cytotoxic function, as well as its relationship with the inflammatory state, is yet to be defined. Here, we characterized the functional profile of polyclonal and HIV-specific CD8^+^ T cells, based on the expression of cell activation and differentiation markers, in individuals chronically infected with HIV, under HAART. Compared with seronegative controls, CD8^+^ T-cells from patients on HAART exhibited a low degranulation capacity (surface expression of CD107a), with consequent low secreted levels and high intracellular expression of granzyme B and perforin. This degranulation defect was particularly observed in those cells expressing the activation marker HLA-DR, which were further characterized as effector memory cells with high expression of CD57. The expression of CD107a, but not of granzyme B and perforin, in CD8^+^ T-cells from HIV-infected patients on HAART reached levels similar to those in seronegative controls when the treatment duration was higher than 25 months. In addition, the expression of CD107a was negatively correlated with the expression of exhaustion markers on CD8^+^ T-cells and the plasma inflammatory molecule sCD14. Thus, despite HAART-induced viral suppression, CD8^+^ T-cells from HIV-infected patients have an alteration in their cytotoxic program. This defect is associated with the cellular activation, differentiation and exhaustion state, as well as with the inflammation levels, and can be partially recovered with a long and continuous treatment.

## Introduction

It is well stablished that CD8^+^ T-cells are critical in the control of HIV infection [1]. Circulating CD8^+^ T-cells are characterized by a high cytotoxic capacity, which depends on both the lytic granule contents (particularly granzymes and perforin), and on the degranulation capacity, widely evaluated by the expression of CD107a [2] [3]. Interestingly, the cytotoxic capacity of circulating CD8^+^ T-cells is modulated according to their differentiation state: i) naïve CD8^+^ T-cells have a low granule content, and a reduced degranulation and cytotoxic capacity; ii) effector and terminally differentiated cells exhibit high cytotoxic capacity; and iii) central memory cells have an intermediate cytotoxic function [4–6]. Importantly, the cytotoxic function of circulating HIV-specific CD8^+^ T-cells has been associated with the control of viral replication and a delayed progression, highlighting the importance of this effector function [7–9].

Since its introduction, the highly active anti-retroviral therapy (HAART) has been effective in preventing deaths related with the acquired immune deficiency syndrome (AIDS) in HIV-infected patients. Nonetheless, partial immune restoration has been observed despite HAART-induced viral suppression [10,11]. Indeed, one of the major pitfalls of anti-retroviral therapy is the persistent immune activation and inflammation, caused by residual viral replication, ongoing co-infections or microbial translocation across a disturbed gastrointestinal mucosa. These defects are associated with the development of non-AIDS conditions [12], such as cardiovascular disease or stroke, which reach up to the 42% of deaths among HIV-infected patients [13]. In addition, an important proportion of deaths in HIV-infected patients on HAART results from a poor immune response against new infectious challenges [13,14].

CD8^+^ T-cells are affected by the increased activation and inflammatory state in HIV-infected patients. These alterations include: (i) persistently increased absolute counts [15]; (ii) increase in the pool of total memory cells with an skewed differentiation [16,17]; (iii) high expression of the activation markers HLA-DR and CD38, and the exhaustion marker Programmed Death (PD)-1 [18,19]; and (iv) low cytotoxic ability [20]. Some of these defects, such as the persistent increase in their absolute counts, activation state and maturation status, are partially reconstituted by HAART [21–23], and, the lack of restoration, are associated with the development of therapeutic failure or non-AIDS conditions [24,25]. Nevertheless, the effect of prolonged HAART-induced viral suppression on the normalization of CD8^+^ T-cells function, particularly their cytotoxic potential, as well as the relation with the activation/differentiation profile and inflammatory state in these individuals, is yet to be defined.

In this study we aimed to evaluate the cytotoxic function and activation/differentiation profile of circulating CD8^+^ T-cells in a cohort of HIV-infected patients on suppressive HAART, as well as the relation with the inflammatory state and treatment duration. We hypothesized that despite HAART-induced viral suppression, a defect in the cytotoxic program was present in CD8^+^ T-cells from HIV-infected patients, and it was directly associated with a high immune activation and inflammation, their differentiation status, and a short treatment duration.

## Materials and methods

### Patients and samples

This study was approved by the Institutional Review Board of Universidad de Antioquia (certificates 15-08-634 and 11-08-352). All experiments followed the principles expressed in the Declaration of Helsinki. All the individuals signed the informed consent. Thirty HIV-infected patients who received only one therapeutic HAART scheme for more than one year, with undetectable viral load (<20 HIV RNA copies/mL) and without previous therapeutic failure, were included from April to December, 2017. A control group of 15 seronegative individuals was also evaluated. The characteristics of the patients are described elsewhere [26]. Notably, similar to previous reports [21–23], compared with seronegative individuals, HIV-infected patients had higher CD8^+^ T-cell counts (median [range] 460 [174–911] vs 737 [268–1250], seronegative vs HIV-infected; P = 0.001), as well as lower CD4:CD8 ratios (median [range] 2 [1.1–2.8] vs 0.7 [0.3–1.5], seronegative vs HIV-infected; P<0.0001). From each individual, 10 mL of venous blood was collected and anticoagulated with EDTA. From the cellular fraction, peripheral blood mononuclear cells were isolated using a Ficoll density gradient (Ficoll Histopaque-1077, Sigma-Aldrich, St. Louis, MO), and washed with RPMI-1640 supplemented with 10% fetal bovine serum, 100 U/mL of penicillin, 100 μg/mL of streptomycin and 2 mM L-glutamine (complete medium) (all from Gibco, Carlsbad, CA). Plasma fraction was used for determining viral load with the clinical diagnostic test RT-PCR Ampliprep-Cobas (Roche, Indianapolis, IN, USA), following the manufacturer’s protocol, with a detection limit of 20 copies/mL. In addition, plasma levels of soluble CD14 (sCD14) were measured using the Human sCD14 ELISA Kit (MyBioSource, San Diego, CA), with a limit of detection of 0.18 ng/mL.

### Evaluation of the phenotype of circulating CD8^+^ T-cells by flow cytometry

One hundred μL of whole blood was incubated for 30 min at room temperature with optimized doses and combinations of the following anti-human antibodies: anti-CD3-PerCP (SK7, BD, San Jose, CA) or anti-CD3-Alexa Fluor 700 (UCHT1, Thermo Fisher, Waltham, MA, USA), anti-CD8-PE (RPA-T8; BD) or anti-CD8-Alexa Fluor 700 (OKT8; Thermo Fisher), anti-CD4-APC-eFluor 780 (RPA-T4, Thermo Fisher), anti-HLA-DR-FITC or APC-eFluor 780 (LN3, Thermo Fisher), anti-CD38-PE Cy7 or PE-eFluor 610 (HIT2, Thermo Fisher), anti-PD-1-PerCP-eFluor 710 (F38-2E2, Thermo Fisher), anti-CCR7-PE (3D12, Thermo Fisher), anti-CD45RO-PE (UCHL1, BD), anti-CD45RA-PE Cy7 (HI100, BD), anti-CD28-PE (L293, BD), anti-CD57-FITC (TB01, Thermo Fisher), anti-CD95-FITC (DX2, BD) and anti-CD152-PE (BNI3, BD Pharmingen). Next, red blood cells were removed with 1X FACS Lysing Solution (BD) during 20 min at room temperature, followed by a washing step with 1 mL of 1X PBS and fixation in 1% paraformaldehyde. The cells were acquired on a LSR Fortessa cytometer (BD), using the FACS Diva software v. 6.0, within one hour of completing the staining; at least 50,000 CD3^+^ events were acquired. Data were analyzed with the FlowJo Software version 10.4 (Tree Star, Inc, Ashland, OR, USA). Fluorescence minus one control was included to define positive thresholds.

### Ex vivo stimulation of CD8^+^ T-cells

Fresh PBMC, at a density of 2x10^6^ cells/mL, were cultured in 96 well V-bottom plates (Costar, Corning, NY). Three different conditions were assessed: 1) cells stimulated only with mouse anti-human CD28 (CD28.2, eBioscience), and CD49d (9F10, eBioscience) functional grade purified antibodies (both at 1 μg/mL), that were used as negative control; 2) cells stimulated with phorbol 12-myristate 13-acetate (PMA), and ionomycin (at 50 and 500 ng/mL, respectively; both from Sigma-Aldrich); 3) cells stimulated with anti-CD28 and anti-CD49d (both at 1 μg/mL) plus a pool of HIV-1 consensus B Gag peptides (at 5 μg/mL) (obtained through the NIH AIDS Reagent Program, Division of AIDS, NIAID, NIH; Cat: 8117, Lot: 140303). All conditions were incubated for the indicated hours at 37°C in 5% CO_2_, in the presence of 5 μg/mL of brefeldin A, and monensin (both from Thermo Fisher), as well as anti-human CD107a-APC (H4A3, BD). After incubation, the levels of cell death were evaluated by the incorporation of the Fixable Viability Dye eFluor 506 (Thermo Fisher) or propidium iodide (PI), and the expression of Annexin V (TACS Annexin V Kits; Trevigen). Next, PBMC were harvested and washed with 2 mL of 1X PBS. Afterwards, lineage antibody cocktail for cell surface staining was added and incubated for 30 min at 4°C, light-protected, followed by a wash, cell fixation and permeabilization with Foxp3/Transcription Factor Staining Buffer Set (Thermo Fisher). Then, proper doses of the following mouse anti-human antibodies were added and incubated for 30 min at 4°C, light-protected: anti-granzyme B-FITC (GB11, BD), anti-perforin-PE (D48, Biolegend, San Diego, CA) and anti-Interferon (IFN)-γ-PE Cy7 (4SB3, Thermo Fisher). Finally, the cells were washed twice with 1X permeabilization solution (Thermo Fisher) and acquired on a LSR Fortessa cytometer (BD). At least 25,000 CD3^+^ CD8^+^ events were acquired. Fluorescence minus one control was also included. Of note, we used an anti–perforin antibody clone that detects both preformed and de novo produced perforin after activation [27,28]. In a fraction of the experiments, cells were cultured in the absence of brefeldin A and monensin, and the supernatants were collected.

### Measurement of granzyme B levels in culture supernatant

The Human granzyme B Flex Set kit (BD) was used for the detection of the respective molecule in supernatants of cultured PBMC, following manufacturer´s protocols. Samples were acquired within an hour following the procedure. The detection limit was 4 pg/mL.

### Ex vivo cytotoxicity assay

The capacity of antigen-specific CD8^+^ T-cells to kill CD4^+^ T-cells, loaded with their cognate peptide was evaluated, as previously described [2,29,30], with some modifications. Briefly, for the preparation of effector CD8^+^ T-cells, fresh autologous PBMC were treated with 5 μg/mL of the HIV-1 Consensus B Gag Peptide Pool, and incubated for 12 hours at 37°C in 5% CO_2_, followed by isolation of CD8^+^ T-cells using the CD8^+^ T-cell Isolation Kit (Miltenyi Biotec, Auburn, CA), with a purity higher than 95%. For the preparation of target cells, CD4^+^ T-cells were isolated with the CD4^+^ T-cell Isolation Kit (Miltenyi Biotec), and labeled with CellTrace CFSE or eBioscience Cell Proliferation Dye eFluor 670 (both from Thermo Fisher), following the manufacturer’s protocols. Cells labeled with CFSE were pulsed for 45 minutes with 5 μg/mL of the pool of HIV-1 consensus B Gag peptides, and then washed three times in complete medium; unloaded CD4^+^ T-cells labeled with eFluor 670 were used as a control. CFSE- and eFluor 670-labeled CD4^+^ T-cells were mixed 1:1 before co-culture. Effector CD8^+^ T-cells and target CD4^+^ T-cells were co-cultured for 6 hours at 37°C in 5% CO_2_ in duplicate in round-bottom 96-well plates in a range of effector to target (E/T) ratios, with a total cellular density of 2x10^6^ cells/mL. The elimination of peptides-pulsed CFSE-labeled cells relative to the unpulsed eFluor 670-labeled cells served as a measure of specific cytotoxicity. CD4^+^ T-cells alone were included to measure basal cell death. After incubation, cells were transferred to FACS tubes, stained with the Fixable Viability Dye eFluor 506 (eBioscience) and lineage antibodies; cells were acquired on the LSR Fortessa cytometer (BD).

### Statistical analysis

GraphPad Prism software v. 7.0 (GraphPad Software, La Jolla, CA) was used for the statistical analysis. Data are presented as medians and ranges, and non-parametric analyses were performed. The Mann-Whitney and Wilcoxon tests were used for comparison of two independent and paired data, respectively. The Kruskal-Wallis and Dunn’s post-hoc tests were used for comparison of three or more groups. The degree of correlation between variables was determined with the Spearman test (rho value). In all cases, a P value <0.05 was considered significant. The Automatic Classification of Cellular Expression by Nonlinear Stochastic Embedding (ACCENSE) analysis was performed to determine the phenotypic relationships from the cell populations evaluated, as we previously described [26]. A Principal Component Analysis (PCA) was performed with the IBM SPSS Statistics software v. 21.

## Results

### Establishing the kinetics of CD8^+^ T-cells activation and degranulation

**Fig 1:**
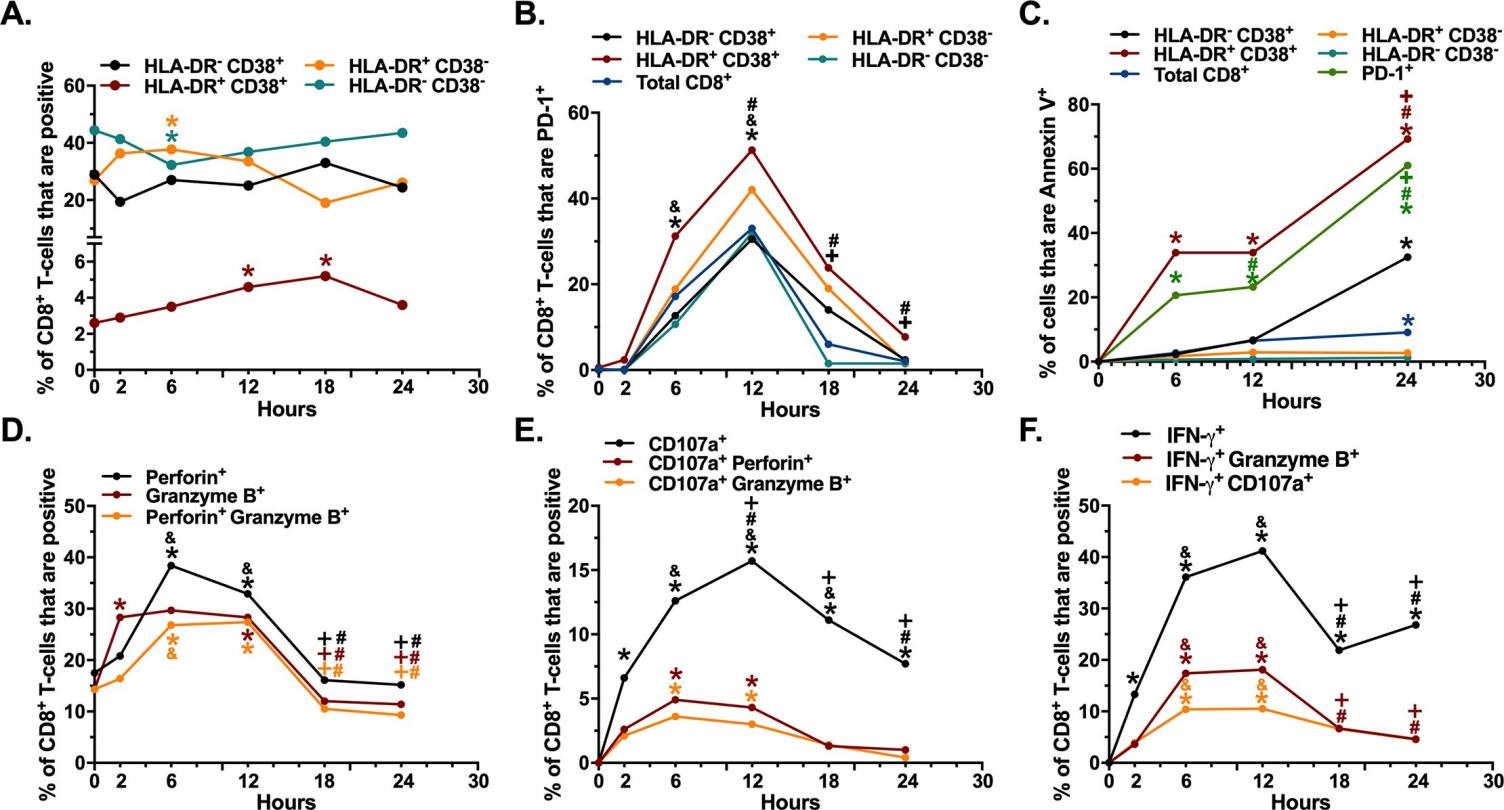
Kinetics of activation of circulating CD8^+^ T-cells. Kinetics of the expression of HLA-DR and CD38 (**A**), PD-1 (**B**), Annexin V (**C**), perforin and/or granzyme B (**D**), CD107a alone or together with granzyme B or perforin (**E**) and IFN-γ alone or together with granzyme B or CD107a (**F**) in CD8^+^ T-cells from seronegative individuals (n = 3) after stimulation with PMA-Ionomycin (at 50 and 500 ng/mL, respectively). In B, the expression of PD-1 in total and HLA-DR/CD38-expressing CD8^+^ T-cells is shown. *P = 0.04 vs 0 hours; &P = 0.04 vs 2 hours; #P = 0.04 vs 6 hours; +P = 0.04 vs 12 hours.

We first characterized, in seronegative individuals, the dynamics of the surface expression of the activation markers HLA-DR and CD38, the exhaustion marker PD-1, the degranulation marker CD107a, and the intracellular expression of perforin, granzyme B and IFN-γ, from 0 to 24 hours after polyclonal stimulation with PMA-Ionomycin, following the gating strategy shown in S1A Fig. At the beginning of the culture, HLA-DR^-^ CD38^-^ cells had the highest proportion (median [range] of 44.4 [41.3–50.1]), followed by HLA-DR^-^ CD38^+^ (median [range] of 28.9 [18.2–29.4]), HLA-DR^+^ CD38^-^ (median [range] of 26.9 [24.1–29]) and HLA-DR^+^ CD38^+^ cells (median [range] of 2.6 [2.4–2.7]) (Fig 1A). As expected, the proportion of HLA-DR^+^ CD38^+^ cells increased from 0 to 18 hours of stimulation, returning to basal levels at 24 hours of stimulation (Fig 1A). The dynamics of HLA-DR^+^ CD38^-^ and HLA-DR^-^ CD38^-^ cells was differential, as the early increase (2–6 hours) of HLA-DR^+^ CD38^-^ cells coincided with a decrease of HLA-DR^-^ CD38^-^ cells, whereas at 18 hours of stimulation the proportion of HLA-DR^+^ CD38^-^ cells decreased, with increase in double negative cells. (Fig 1A). Finally, there were no changes in the proportion of HLA-DR^-^ CD38^+^ cells (Fig 1A).

Consistent with previous reports [31,32], the expression of PD-1 increased after 6 hours of stimulation, with a peak at 12 hours (Fig 1B), followed by a decrease in their levels after 18 hours of culture (Fig 1B). This pattern was also observed in HLA-DR/CD38-expressing CD8^+^ T-cells, with the highest expression of PD-1 in HLA-DR^+^ CD38^+^ cells (Fig 1B), consistent with their activated phenotype. Finally, cell death was evaluated by the expression of Annexin V and the internalization of PI. Of note, a low to undetectable frequency of cells were PI^+^ (S1A Fig), so that only the frequency of Annexin V^+^ cells is reported. Under this analysis, as expected, PD-1^+^ and HLA-DR^+^ CD38^+^ CD8^+^ T-cells had the higher levels of cell death, which increased after 6–12 hours of culture (Fig 1C), consistent with a lower expression of anti-apoptotic molecules of these subsets [33,34]. In addition, the frequency of Annexin V^+^ HLA-DR^-^CD38^+^ CD8 ^+^ T-cells increased at 24 hours after stimulation. However, the viability of total CD8^+^ T-cells, as well as HLA-DR^+^ CD38^-^ and HLA-DR^-^ CD38^-^ cells remained higher than 90% throughout the culture (Fig 1C). Of note, in the unstimulated cells control, the frequencies of HLA-DR/CD38/PD-1-expressing cells, as well as those of dead cells, where similar to those seen in stimulated cells at 0 hours, and remained constant at all time points (S1B–S1D Fig).

When we evaluated the intracellular expression of cytotoxic molecules, at 0 hours of culture, an important proportion of CD8^+^ T-cells expressed granzyme B and perforin when they were measured independent or simultaneously (Fig 1D). Similar to previous reports [2,28,35], there was a rapid up-regulation of both cytotoxic molecules between 2 and 6 hours of stimulation, followed by a decrease after 12 hours (Fig 1D), consistent with their release after cell degranulation [2,35]. In addition, the expression of CD107a continuously increased from 2 to 12 hours of culture, with a subsequent decrease (Fig 1E). Moreover, an important fraction of CD107a^+^ cells co-expressed perforin or granzyme B, indicative of de novo production of these cytotoxic molecules [28] (Fig 1E). Finally, the expression of IFN-γ, alone or together with granzyme B and CD107a continuously increased from 2 to 12 hours of culture, followed by a decrease in the proportion of these cells after 12 hours of culture (Fig 1F). As expected, there were no changes in the expression of granzyme B and perforin in unstimulated cells (S1E Fig), with undetectable levels of CD107a and IFN-γ at all time points.

Finally, we evaluated the kinetics of expression of HLA-DR, CD38, PD-1, effector molecules, and cell viability (Fixable Viability Dye), in cells from HIV-infected individuals. As shown in S2A Fig, there was an increase in HLA-DR^+^ CD38^+^ and HLA-DR^-^ CD38^+^ CD8^+^ T-cells and decrease in HLA-DR^-^ CD38^-^ cells after 12 hours of stimulation. The pattern and kinetics of PD-1 expression was similar to that of seronegative individuals, with an increase after 6 hours of stimulation, peak at 12 hours, and a decrease after 18 hours of culture (S2B Fig). Similarly, HLA-DR^+^ CD38^+^ CD8^+^ T-cells exhibited the highest levels of PD-1 (S2B Fig). Moreover, HLA-DR^+^ CD38^+^ and PD-1^+^ CD8^+^ T-cells had the highest levels of cell death, which increased after 6–12 hours of culture (S2C Fig). In addition, total CD8^+^ T-cells from HIV-infected patients had higher levels of cell death at 12 and 24 hours of culture, in comparison with cells from seronegative individuals (P = 0.04). Ultimately, the expression of perforin, granzyme B, CD107a and IFN-γ in CD8^+^ T-cells from HIV-infected patients followed a similar kinetics than in cells from seronegative individuals, with the peak of degranulation, decrease of cytotoxic molecules, and production of IFN-γ occurring at 12 hours of culture (S2D–S2F Fig). Of note, there were no changes in the expression of phenotypic and functional markers in unstimulated cells throughout culture (P≥0.4). Together, these results confirm that the stimulation with PMA-ionomycin induces the activation of CD8^+^ T-cells, with most of the phenotypic and functional changes reaching a peak at 12 hours of culture. Therefore, this was the time point chosen for subsequent experiments.

### Low degranulation capacity in CD8^+^ T-cells from HIV-infected patients despite HAART-induced viral suppression

**Fig 2:**
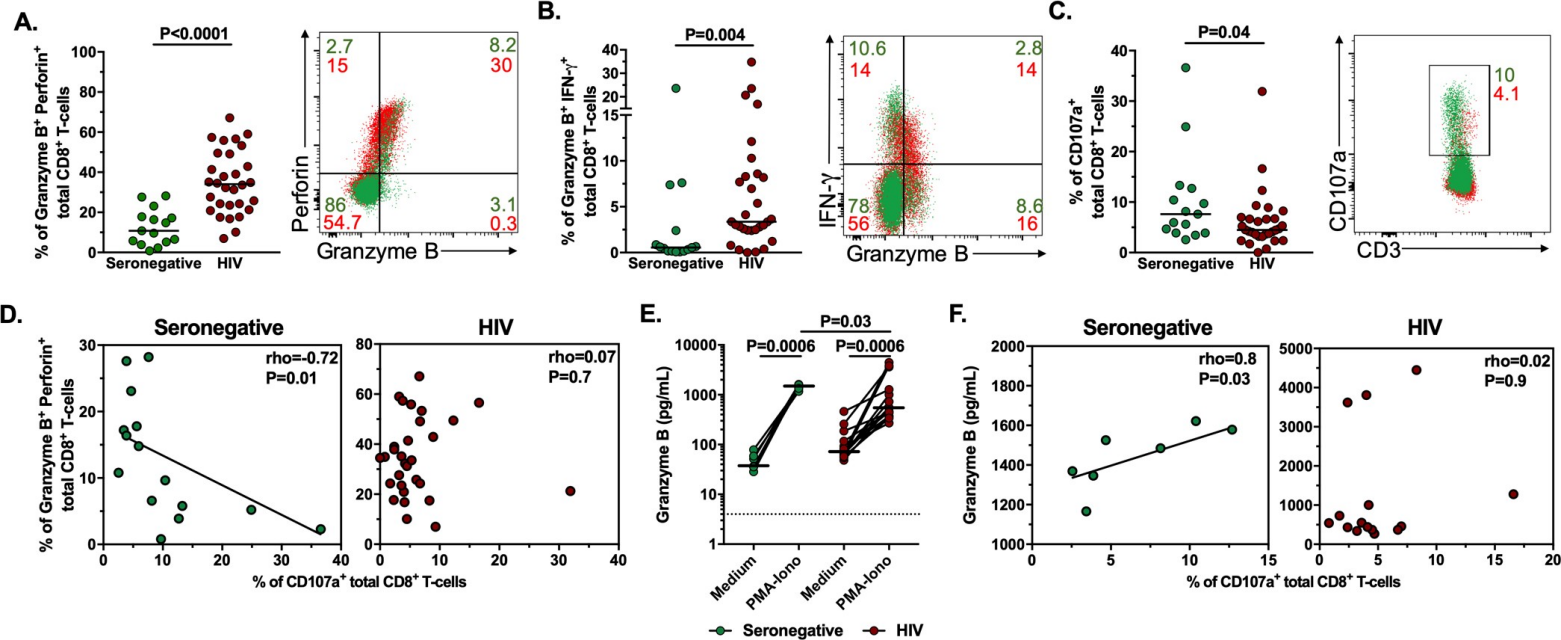
Alteration in the cytotoxic program of CD8^+^ T-cells from HIV-infected patients. Frequencies of granzyme B^+^ perforin^+^ (**A**), granzyme B^+^ IFN-γ^+^ (**B**) and CD107a^+^ (**C**) CD8^+^ T-cells in seronegative (n = 15) and HIV-infected (n = 30) individuals after polyclonal stimulation. **D**. Correlation between the frequency of granzyme B^+^ perforin^+^ and CD107a^+^ CD8^+^ T-cells in seronegative and HIV-infected individuals (n = 15 and n = 30, respectively). **E**. Levels of granzyme B in supernatant of cultured PBMC from seronegative and HIV-infected individuals (n = 7 and n = 15, respectively). The dashed line indicates the assay limit of detection. **F.** Correlation between the levels of granzyme B in supernatant of cultured PBMC and the frequency of CD107a^+^ CD8^+^ T-cells in seronegative and HIV-infected individuals after polyclonal stimulation (n = 7 and n = 15, respectively). In A-C and E, the P value of the Mann-Whitney test is shown. In D and F, the rho and P value of the Spearman test are shown.

To determine if there is any alteration in the cytotoxic program of CD8^+^ T-cells from HIV-infected patients under suppressive HAART, we evaluated the expression of cytotoxic molecules and the degranulation of total CD8^+^ T-cells from HIV-infected patients compared with seronegative individuals. Similar to previous reports [36,37], CD8^+^ T-cells from HIV-infected patients exhibited a higher frequency of granzyme B^+^ perforin^+^ cells after polyclonal stimulation (indicative of the remaining cytotoxic molecules in granules after cell degranulation; Fig 2A), but not in unstimulated cells (indicative of preformed stored molecules; S3A Fig), compared to seronegative individuals. HIV-infected patients also had a higher frequency of CD8^+^ T-cells expressing granzyme B^+^ alone (S3B Fig), but in the case of granzyme B^-^ perforin^+^ cells, the frequency was lower in HIV-infected patients (S3B Fig), indicating that most of perforin is co-expressed with granzyme B in CD8^+^ T-cells in these individuals. Consequently, the frequency of granzyme B^-^ perforin^-^ cells was lower in HIV-infected patients (S3B Fig). In addition, the frequency of granzyme B^+^ IFN-γ^+^ (indicative of cell activation, Fig 2B) CD8^+^ T-cells was also increased in HIV-infected patients, suggestive of a pre-activation status of CD8^+^ T-cells, as we previously reported for HIV-infected patients on HAART [26].

Strikingly, we additionally found a lower frequency of total CD107a^+^ CD8^+^ T-cells in HIV-infected patients compared to uninfected individuals (Fig 2C). This result was not found for CD107a^+^, granzyme B^+^ or CD107a^+^ perforin^+^ cells after polyclonal stimulation (S3C Fig). Since the co-expression of granzyme B or perforin and CD107a is indicative of de novo production and granule recruitment of the cytotoxic molecules [28], these results suggest an alteration in the bulk degranulation ability of CD8^+^ T-cells in HIV-infected individuals, but not in the de novo production of cytotoxic molecules [28]. Considering that previous reports [2,35] and our kinetic studies (Fig 1D and 1E) demonstrate the opposing pattern of expression of intracellular cytotoxic molecules and surface CD107a^+^ after stimulation of CD8^+^ T-cells (indicative of release of the granule content with degranulation), the correlation between the frequency of granzyme B^+^ perforin^+^ and that of CD107a^+^ CD8^+^ T-cells was evaluated. As shown in Fig 2D, in contrast to the seronegative group (left panel), this negative correlation was not observed for HIV-infected patients (right panel). Moreover, we evaluated the levels of granzyme B in culture supernatant from PBMC from seronegative and HIV-infected individuals. Although these levels were higher in cells treated with PMA-ionomycin compared with unstimulated cells in both groups (Fig 2E), the levels were lower in stimulated cells from HIV-infected patients compared with those from seronegative controls (Fig 2E). Finally, the correlation between the levels of granzyme B in culture supernatant and the frequency of CD107a^+^ CD8^+^ T-cells after polyclonal stimulation, was evaluated. A positive correlation was found in seronegative individuals (Fig 2F left panel), consistent with the release of granzyme B with degranulation, but there was no correlation in HIV-infected patients (Fig 2F right panel). Thus, CD8^+^ T-cells from HIV-infected patients have an alteration in their cytotoxic program after polyclonal stimulation, with low degranulation capacity and consequent increased intracellular expression of cytotoxic molecules, but decreased in culture supernatant, which would limit their content in a potential immunological synapse.

### HLA-DR^+^ subsets are effector memory cells and exhibit the major cytotoxic potential among CD8^+^ T-cells; a shift in the differentiation program of CD8^+^ T-cells from HIV-infected patients

**Fig 3:**
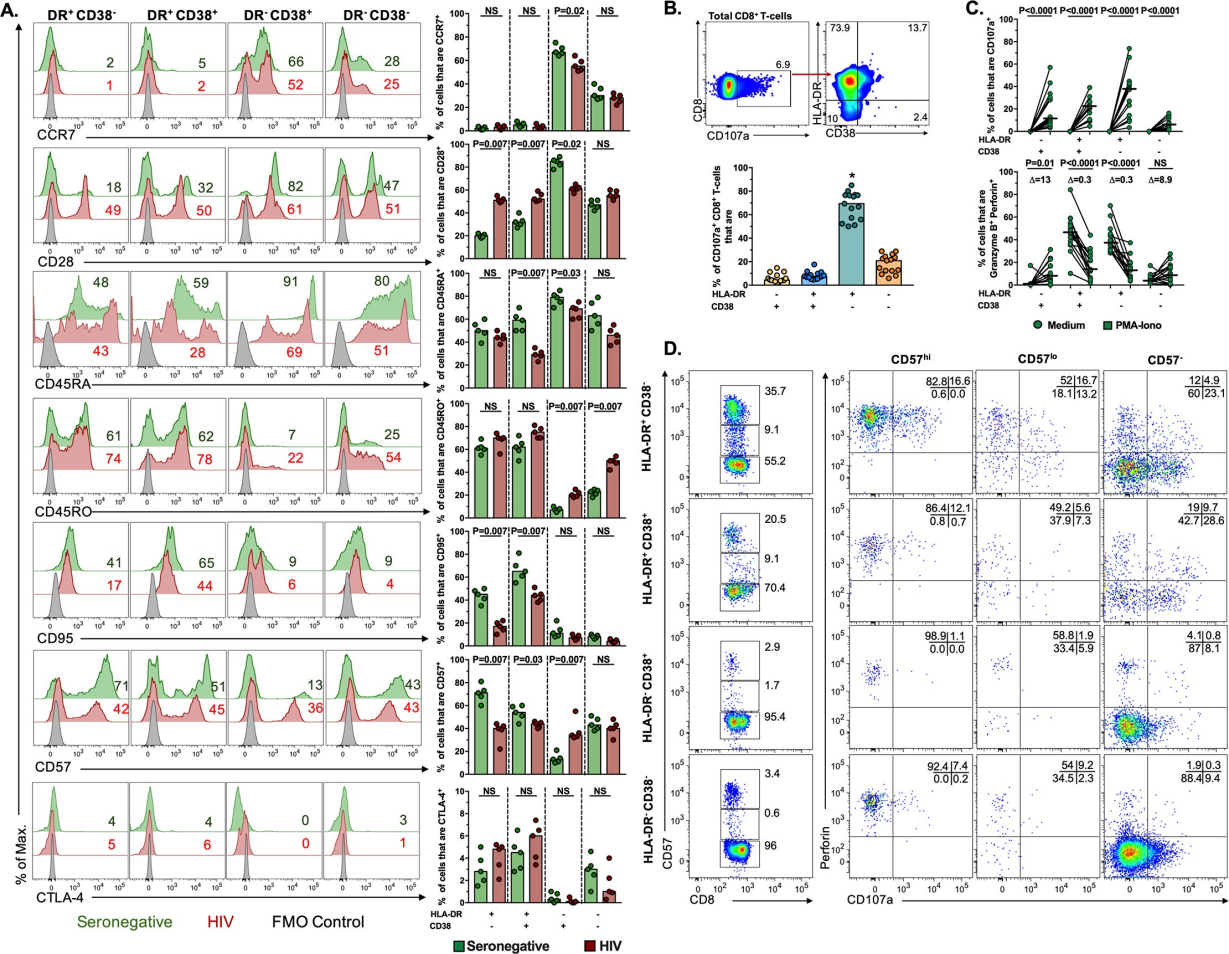
HLA-DR^+^ CD8^+^ T-cells exhibit an effector memory profile. **A**. Expression of CCR7, CD28, CD45RA, CD45RO, CD95, CD57, and CTLA-4 in HLA-DR/CD38-expressing CD8^+^ T-cells from a representative seronegative and HIV-infected individual. The numbers indicate the percentage of positive cells for each marker. The summary of the results is shown in the right panels (n = 5 in both groups of individuals). **B.** Expression of HLA-DR and CD38 in CD107a^+^ CD8^+^ T-cells from a representative seronegative individual after 12 hours treatment with PMA-ionomycin. The summary of the results is shown in the lower panel (n = 15). *P≤0.01 vs all other subsets. **C**. Frequencies of HLA-DR/CD38-expressing CD8^+^ T-cells that are CD107a^+^ (upper panel), and granzyme B^+^ perforin^+^ (lower panel), in seronegative individuals (n = 15). The P value of the Wilcoxon test is shown. **D.** Expression of perforin and CD107a in CD57^hi^, CD57^lo^ and CD57^-^ cells among HLA-DR/CD38-expressing CD8^+^ T-cells from a representative seronegative individual after PMA-ionomycin stimulation, from a total of 5 individuals. NS: Not statistically significant.

Next, we evaluated the cytotoxic program according to the differential expression of HLA-DR and CD38 in CD8^+^ T-cells, whose co-expression has been associated with an activated and dysfunctional state of CD8^+^ T-cells [33]. First, we phenotypically characterized the differentiation state of HLA-DR/CD38-expressing CD8^+^ T-cells subsets, for exploring an association between the phenotype and the cytotoxic potential. Thus, we evaluated the expression of the differentiation markers CCR7, CD28, CD45RA, CD45RO, CD95, CD57 and Cytotoxic T-lymphocyte Antigen 4 (CTLA-4) in each subset, in resting peripheral blood. As shown in Fig 3A, in seronegative individuals, the expression of HLA-DR was associated with an effector memory phenotype, with high frequency of CD45RO, CD95 and CD57 positive cells, and low frequency of CCR7, CD28 and CTLA-4-positive cells in the HLA-DR^+^ subsets. On the contrary, the expression of CD38 was associated with a resting (naïve) phenotype, characterized by high expression of CD45RA, CCR7 and CD28 [38]. The HLA-DR^-^ CD38^-^ cells had a mixed phenotype. When compared with seronegative controls, HLA-DR^+^ CD38^-/+^ cells from HIV-infected patients exhibited a shift to a less differentiated phenotype, given the higher proportion of cells expressing CD28 and the lower proportion of cells expressing CD57 and CD95. On the contrary, the HLA-DR^-^ CD38^+^ subset had a shift to a more differentiated phenotype in HIV-infected patients, exhibiting a lower proportion of cells expressing CCR7, CD28 and CD45RA cells, and an increase in the proportion of cells expressing CD45RO and CD57. Moreover, we focused our analyses on the basal expression of CCR7 and CD45RA, which define the naïve (CCR7^+^ CD45RA^+^), central memory (CM; CCR7^+^ CD45RA^-^), effector memory (EM; CCR7^-^ CD45RA^-^), and EM which express CD45RA (TEMRA; CCR7^-^ CD45RA^+^) CD8^+^ T-cells [39] (S4A Fig). Similar to previous reports [16,40], HIV-infected patients exhibited higher frequencies of CM and TEMRA cells, and lower frequencies of naïve cells, compared with seronegative individuals; the frequencies of EM cells were comparable between both groups (S4A Fig). In seronegative individuals, most of HLA-DR^+^ CD38^-/+^ CD8^+^ T-cells displayed a TEMRA and/or EM phenotype, whereas HLA-DR^-^ CD38^+^ CD8^+^ T-cells were mainly naïve cells (S4B Fig). According to the results of Fig 3A, HLA-DR^+^ CD38^-/+^ CD8^+^ T-cells from HIV-infected patients exhibited a decrease in the proportion of TEMRA and EM cells, but increase in CM and naïve cells (S4B Fig). On the other hand, HLA-DR^-^ CD38^+^ CD8^+^ T-cells from HIV-infected patients had a lower proportion of naïve cells but increase in TEMRA and EM cells (S4B Fig). Similarly, when analyzing the expression of HLA-DR and CD38 in the four CCR7/CD45RA-expressing subsets of CD8^+^ T-cells, most of TEMRA and EM cells were HLA-DR^+^ CD38^-/+^ in seronegative individuals, but the CD38^+^ subsets were enriched in HIV-infected patients (S4C Fig). In addition, in seronegative individuals, most of CM cells were HLA-DR^-^ CD38^-^, followed by HLA-DR^+^ CD38^-^, but this pattern was inverted in HIV-infected patients (S4C Fig). Finally, most of naive cells were HLA-DR^-^ CD38^-^ or HLA-DR^-^ CD38^+^ in seronegative individuals, but there was an increase in HLA-DR^+^ CD38^-^ and decrease in HLA-DR^-^ CD38^-^ cells in HIV-infected patients (S4C Fig). The findings are summarized in Table 1. Altogether, these results indicate that, in seronegative individuals, HLA-DR and CD38 characterize late- and early-differentiated CD8^+^ T-cells, respectively. Although CD38 has been referred as an activation marker [24,41], this is true only when is co-expressed with HLA-DR. These results are consistent with the expression of CD38 by T-cells with a naïve phenotype and thymocytes [42,43], and the high frequency of HLA-DR^-^ CD38^+^ T-cells in the early stages of life [44]. Moreover, our results indicate that, in HIV-infected patients, there is a shift to a less differentiated profile in HLA-DR-expressing CD8^+^ T-cells, and a shift to a more differentiate state in CD38-expressing cells, consistent with the redistribution of memory CD8^+^ T-cell subsets during this infection [16,40].

**Table 1:** Expression of CD8^+^ T-cell memory and functional markers in HLA-DR/CD38-expressing cells.

Marker	Expression in CD8^+^ T-cell subsets from seronegative individuals				Expression in CD8^+^ T-cell subsets in HIV-infected patients relative to seronegative individuals			
	DR^+^ CD38^-^	DR^+^ CD38^+^	DR^-^ CD38^+^	DR^-^ CD38^-^	DR^+^ CD38^-^	DR^+^ CD38^+^	DR^-^ CD38^+^	DR^-^ CD38^-^
**CCR7**	-	-	+++	+	=	=	↓	=
**CD28**	+	+	+++	++	↑	↑	↓	=
**CD45RA**	+	+	+++	++	=	↓	↓	=
**CD45RO**	++	++	-	+	=	=	↑	↑
**CD95**	+	++	-/+	-/+	↓	↓	=	=
**CD57**	+++	++	-/+	+	↓	↓	↑	=
**CTLA-4**	-	-	-	-	=	=	=	=
**CD107a**^**1**^	+++	++	+	+	↓	↓	↓	=
**Granzyme B/Perforin**^**2**^	+++	+++	-	+	↑	↑	↑	↑
**IFN-γ**^**1**^	+++	+++	+	++	↑	↑	↑	↑
**Defining phenotype**	Effector memory		Naive	Mixed phenotype	Shift to a less differentiated state		Shift to a more differentiated state	

^1^ After PMA-ionomycin stimulation.

^2^ In basal conditions.

Next, we evaluated the cytotoxic potential of the HLA-DR/CD38-expressing subsets after polyclonal stimulation. As shown in Fig 3B, in seronegative individuals, most of CD107a^+^ CD8^+^ T-cells expressed the HLA-DR^+^ CD38^-^ phenotype. Consistent with this result, when the proportions of CD107a^+^ and granzyme B^+^ perforin^+^ cells were evaluated, the HLA-DR^+^ subsets (both CD38^+^ and CD38^-^) exhibited the highest degranulation and decrease in the expression of cytotoxic molecules (Fig 3C). In contrast, with stimulation, HLA-DR^-^ CD38^+^ and HLA-DR^-^ CD38^-^ cells had a low expression of CD107a (Fig 3B), whereas had an increase in the co-expression of granzyme B and perforin (Fig 3C). No significant differences between HLA-DR/CD38-expressing subsets were found for granzyme B/IFN-γ co-expression after stimulation (P≥0.1). Furthermore, we evaluated the expression of granzyme B and IFN-γ in CCR7/CD45RA-expressing CD8^+^ T-cells after PMA-ionomycin stimulation. Similar to previous reports [4,5], TEMRA and EM cells from seronegative individuals exhibited the highest expression of granzyme B among CD8^+^ T-cells, followed by CM and naïve cells (S5 Fig). The production of IFN-γ was also dominated by memory subsets, compared with naïve cells (S5 Fig). Remarkably, naïve cells from HIV-infected patients had a higher expression of granzyme B and IFN-γ after stimulation than those from seronegative individuals, whereas an opposite pattern was found for CM and EM cells (S5 Fig). These results are in agreement with our phenotypic characterization of HLA-DR/CD38-expressing memory subsets (Fig 3A and S4 Fig), showing a more mature profile of naïve CD8^+^ T-cells and a skewed maturation of CM and EM subsets.

CD57 expression in CD8^+^ T-cells has been associated with the levels of cytotoxic molecules [5,45]. Thus, we evaluated the expression of CD57 in total and HLA-DR/CD38-expressing CD8^+^ T-cells after polyclonal stimulation, along with the expression of perforin and CD107a. CD57^hi^, CD57^lo^ and CD57^-^ cells were identified in seronegative individuals, based on at least 1 log difference in the median fluorescence intensity of the populations (S6A Fig). According to a previous report [5], almost all CD57^hi^ CD8^+^ T-cells were perforin^+^, whereas CD57^lo^ and CD57^-^ cells had an intermediate and low expression of this cytotoxic molecule, respectively (S6A Fig). CD57^hi^ and CD57^lo^ cells exhibited a comparable expression of CD107a, and it was higher than in CD57^-^ cells (S6A Fig). In addition, after polyclonal stimulation, HLA-DR^+^ CD38^-^ CD8^+^ T-cells exhibited the highest proportion of CD57^hi^ cells, followed by HLA-DR^+^ CD38^+^, HLA-DR^-^ CD38^-^ and HLA-DR^-^ CD38^+^ cells (Fig 3D); these CD57-expressing subsets exhibited a pattern of expression of perforin and CD107a similar to that of total CD8^+^ T-cells (Fig 3D). Collectively, our phenotypic and functional characterization of CD8^+^ T-cell subsets indicate that HLA-DR-expressing cells are an effector memory subset with the highest activation of the cytotoxic program after polyclonal activation among CD8^+^ T-cells. In HIV-infected patients, despite HAART-induced viral suppression, late-differentiated CD8^+^ T-cells have a skewed maturation [17], whereas early-differentiated CD8^+^ T-cells have a more mature profile [46].

### HLA-DR^+^ CD8^+^ T-cells have the major alterations in the cytotoxic program in HIV-infected patients

**Fig 4:**
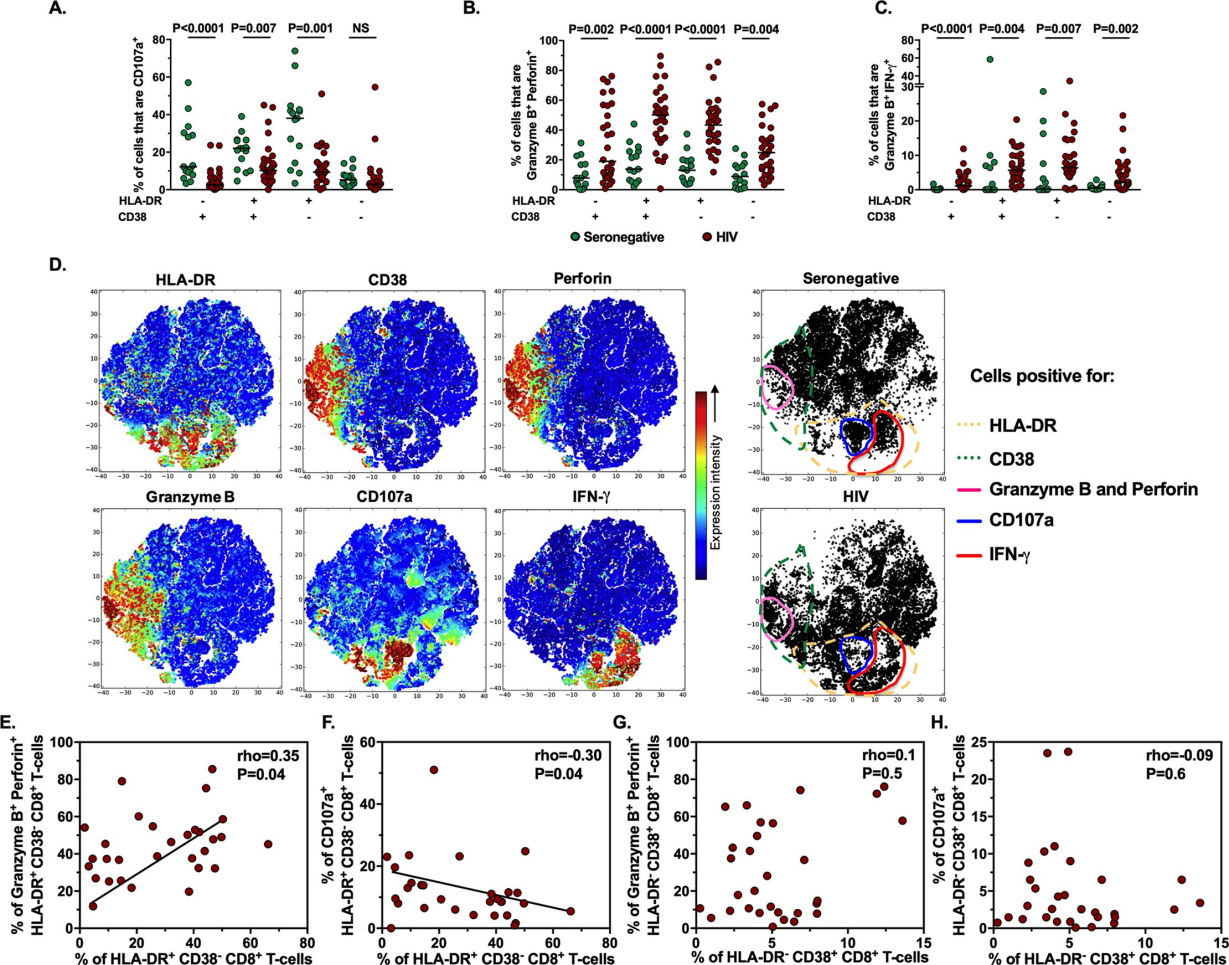
Alteration in the cytotoxic program of HLA-DR/CD38-expressing CD8^+^ T-cells from HIV-infected patients. Frequencies of HLA-DR/CD38-expressing CD8^+^ T-cells that are CD107a^+^ (**A**), granzyme B^+^ perforin^+^ (**B**) and granzyme B^+^ IFN-γ^+^ (**C**) in seronegative (n = 15) and HIV-infected (n = 30) individuals. The P value of the Mann-Whitney test is shown. NS: Not statistically significant. **D**. Cell ACCENSE t-SNE plots of HLA-DR, CD38, perforin, granzyme B, CD107a and IFN-γ expression in merged stimulated CD8^+^ T-cells from representative seronegative and HIV-infected individuals (n = 4 in each group; M = 60,000 cells). The circles indicate the cells positive for the indicated molecules. **E-H.** Correlation between the frequency of granzyme B^+^ perforin^+^ or CD107a^+^ cells and the frequency of their respective total HLA-DR^+^ CD38^-^ (**E** and **F**) or HLA-DR^-^ CD38^+^ (**G** and **H**) CD8^+^ T-cells from HIV-infected individuals (n = 30). The rho and P value of the Spearman test are shown.

Since total CD8^+^ T-cells from HIV-infected patients exhibited an alteration in their cytotoxic program, we compared the cytotoxic potential of HLA-DR/CD38-expressing CD8^+^ T-cells between seronegative and HIV-infected individuals. Similar to what was found for total CD8^+^ T-cells, HLA-DR/CD38-expressing cells from HIV-infected patients showed a lower proportion of CD107a^+^ cells (Fig 4A, and S6B and S6C Fig), and a higher proportion of granzyme B^+^ perforin^+^ and granzyme B^+^ IFN-γ^+^ cells (Fig 4B and 4C) compared with seronegative donors. Of note, these alterations were particularly observed in HLA-DR^+^ CD38^+/-^ cells, suggesting that the defect in the cytotoxic program is associated with the expression of HLA-DR. These findings were extended with a t-SNE analysis of merged stimulated CD8^+^ T-cells from seronegative and HIV-infected individuals (Fig 4D). Of note, the t-SNE analysis allows to determine the phenotypic relationships from the cell populations evaluated [47]. Compared with seronegative controls, HLA-DR- and CD38-expressing cells were more abundant in HIV-infected patients, and they were characterized by a higher expression of granzyme, perforin and IFN-γ, but a lower expression of CD107 (Fig 4D), the latter particularly observed in HLA-DR-positive cells. Strikingly, in HIV-infected patients, the frequencies of HLA-DR^+^ CD38^-^ CD8^+^ T-cells positively correlated with their respective proportions of granzyme B^+^ perforin^+^ cells (Fig 4E), but negatively correlated with the frequency of CD107a^+^ cells (Fig 4F). These associations were not found for the HLA-DR^-^ CD38^+^ (Fig 4G and 4H). In summary, HLA-DR-expressing CD8^+^ T-cells from HIV-infected patients exhibit the main alterations in their cytotoxic program.

### Most of HIV-specific CD8^+^ T-cells are effector HLA-DR^+^ cells

**Fig 5:**
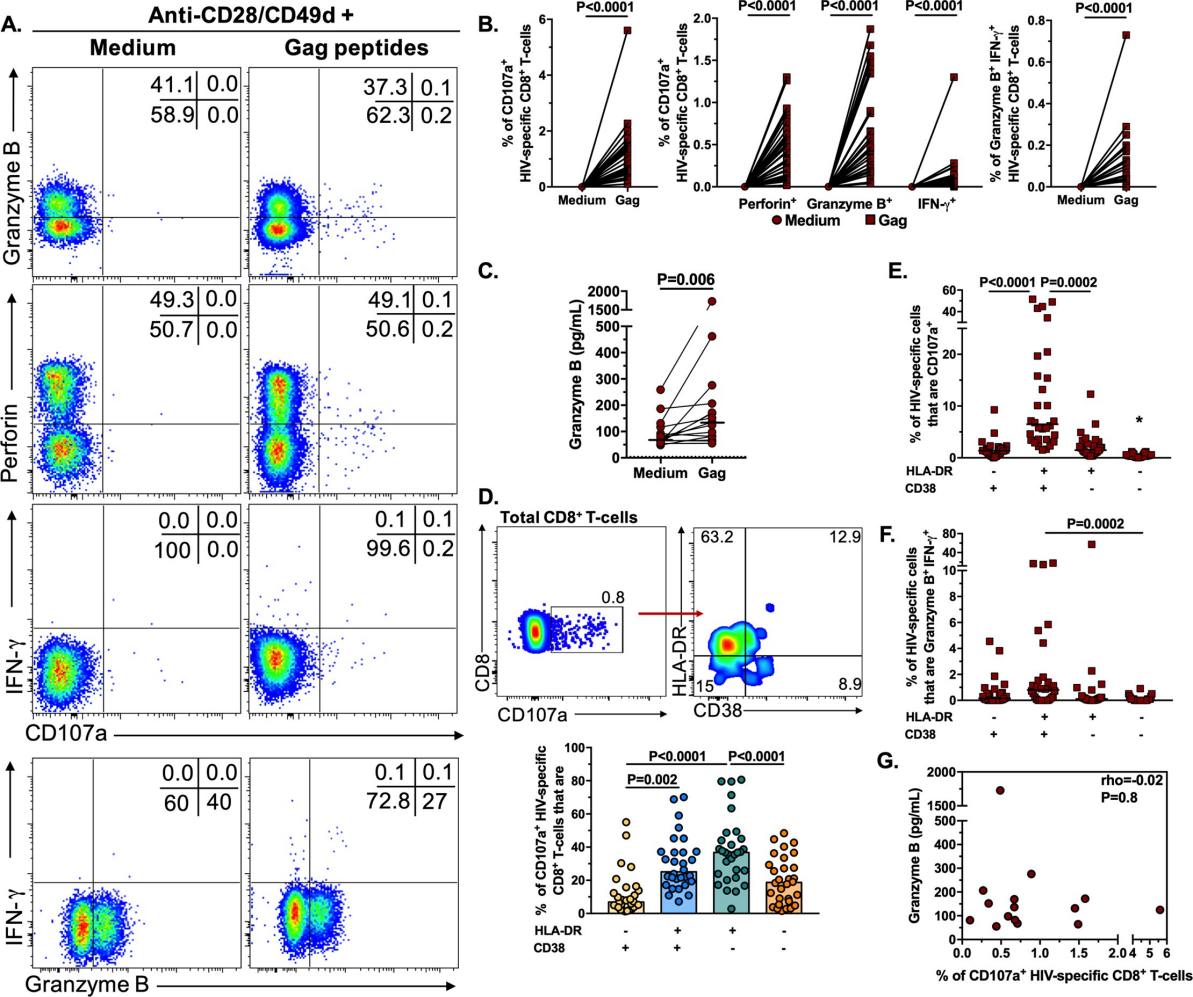
Cytotoxic program of HIV-specific CD8^+^ T-cells. **A-B.** Expression of CD107a alone or together with perforin, granzyme B or IFN-γ, and granzyme B plus IFN-γ in CD8^+^ T-cells treated with medium or a pool of HIV Gag peptides (n = 30). The P value of the Wilcoxon test is shown. **C**. Levels of granzyme B in supernatant of cultured unstimulated or Gag peptides-stimulated PBMC from HIV-infected individuals (n = 15). The dashed line indicates the assay limit of detection. **D**. Expression of HLA-DR and CD38 in CD107a^+^ CD8^+^ T-cells from a representative HIV-infected individual, after stimulation with a pool of HIV Gag peptides. The summary of the results is shown in the lower panel (n = 30). **E-F.** Frequencies of HLA-DR/CD38-expressing CD8^+^ T-cells that are CD107a^+^ (**E**), and granzyme B^+^ IFN-γ^+^ (**F**) in HIV-infected individuals (n = 30), after stimulation with a pool of HIV Gag peptides. **G**. Correlation between the levels of granzyme B in supernatant of cultured PBMC and the frequency of CD107a^+^ CD8^+^ T-cells in HIV-infected individuals (n = 15) after stimulation with a pool of HIV Gag peptides. The rho and P value of the Spearman test are shown. In D, E and F, the P value of the Dunn’s post-hoc test is shown.

To determine if, similar to total CD8^+^ T-cells, HIV-specific CD8^+^ T-cells also exhibit an alteration in their cytotoxic potential, we characterized the expression of cytotoxic molecules and degranulation of total and HLA-DR/CD38-expressing CD8^+^ T-cells from HIV-infected patients after stimulation with a pool of HIV Gag peptides. The proportion of CD107a^+^ cells, alone or co-expressed with granzyme B, perforin or IFN-γ, as well as granzyme B^+^ IFN-γ^+^ cells, significantly increased after Gag stimulation (Fig 5A and 5B). The proportion of granzyme B^+^ perforin^+^ cells did not significantly change after stimulation (S6D Fig), but the supernatant levels of granzyme B were higher in Gag-stimulated cells compared with unstimulated cells (Fig 5C). Similar to that found in cells from seronegative individuals stimulated with PMA-ionomycin, most of the HIV-specific CD107a^+^ CD8^+^ T-cells from HIV-infected individuals expressed the HLA-DR^+^ CD38^-^ phenotype (Fig 5D), and these cells had the similar pattern of expression of CD57 and perforin, with CD57hi cell expressing the highest levels of the cytotoxic molecule (S7 Fig). In addition, HLA-DR^+^ CD38^+^ cells exhibited the highest proportion of CD107a^+^, and granzyme B^+^ IFN-γ^+^ cells (Fig 5E and 5F). Ultimately, to determine if there was an alteration in the cytotoxic program of HIV-specific CD8^+^ T-cells, we explored the correlation between the levels of degranulation (frequency of CD107a^+^ HIV-specific CD8^+^ T-cells), and those of granzyme B in culture supernatant, both in cells stimulated with Gag peptides. Similar to what we found for total CD8^+^ T-cells (Fig 2F), we did not find a correlation between those parameters (Fig 5G), suggesting that the degranulation capacity, and consequent release of cytotoxic molecules, is also affected in HIV-specific CD8^+^ T-cells. Thus, HIV-specific CD8^+^ T-cells, evaluated by the activation of their cytotoxic program, are particularly constituted by HLA-DR^+^ cells, and apparently exhibit similar alterations to those of total CD8^+^ T-cells.

### A longer HAART duration is associated with recovery of the cytotoxic program of CD8^+^ T-cells from HIV-infected patients

**Fig 6:**
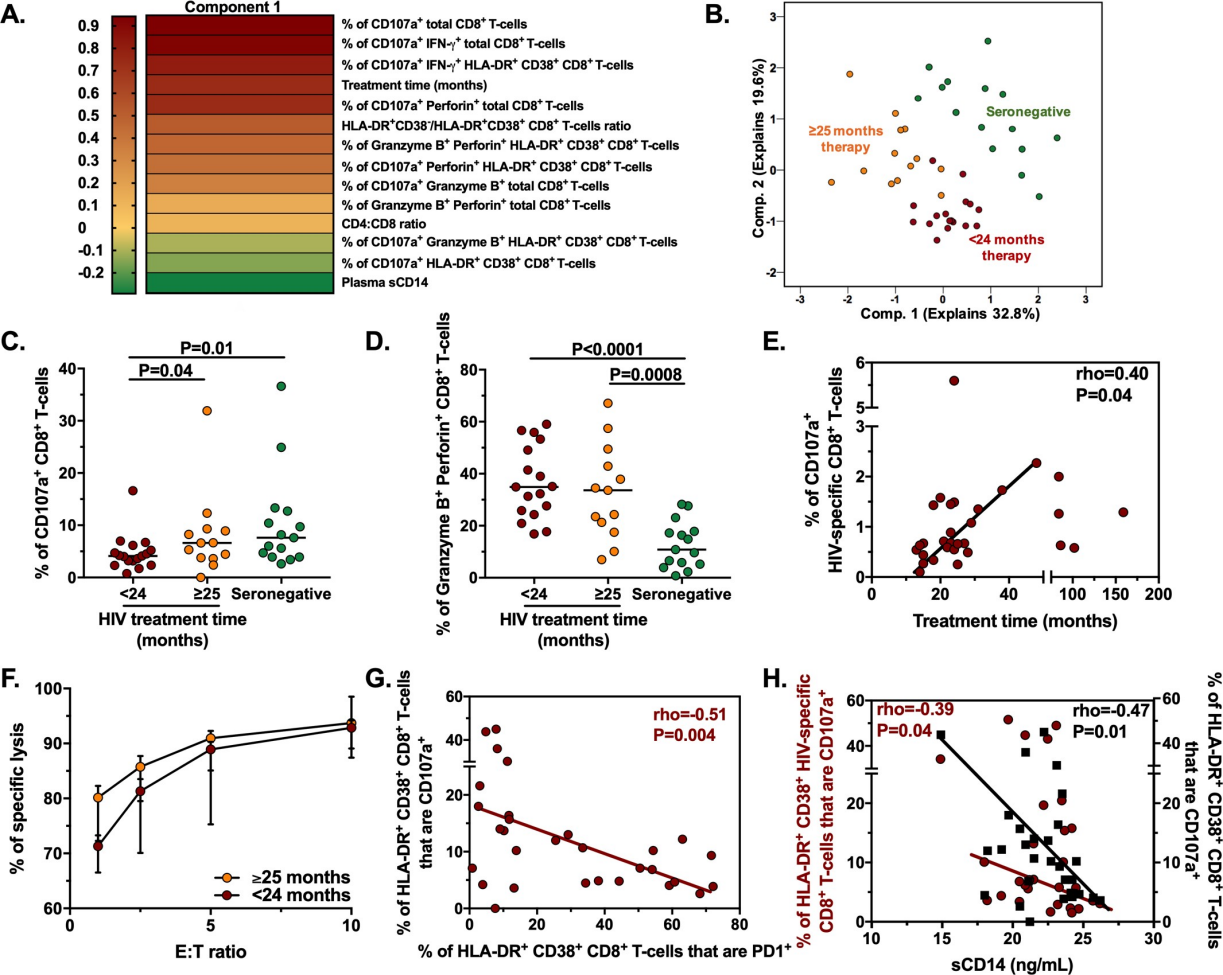
Association between the treatment duration, the exhaustion/activation state and inflammation levels with the cytotoxic program of CD8^+^ T-cells from HIV-infected patients. **A.** Heat map of the weight of the variables included in the Component 1 of the Principal Component Analysis performed, including the phenotypic and functional characterization of CD8^+^ T-cells, as well as other variables such as the length of the treatment, the CD4:CD8 ratio, and plasma levels of sCD14. **B**. Spatial distribution of seronegative (n = 15) and HIV-infected individuals, the latter classified in those with <24 months (n = 17) or ≥25 (n = 13) months of therapy, after Principal Component Analysis. **C-D**. Frequencies of CD107a^+^ (**C**) and granzyme B^+^ perforin^+^ (**D**) CD8^+^ T-cells in seronegative (n = 15) and HIV-infected individuals, the latter classified in those with <24 months or ≥25 months of therapy (n = 17 and n = 13, respectively). **E**. Correlation between the frequency of CD107a^+^ CD8^+^ T-cells after Gag peptides stimulation and the treatment time in months in HIV-infected patients (n = 30). **F**. Percentage of specific lysis after in vitro cytotoxicity assay in cells from HIV-infected patients with <24 or ≥25 months of therapy (n = 3 in each group, run in duplicate in each case). **G**. Correlation between the frequency of HLA-DR^+^ CD38^+^ CD8^+^ T-cells that are CD107a^+^ after PMA-ionomycin stimulation and that of HLA-DR^+^ CD38^+^ CD8^+^ T-cells that are PD-1^+^ in resting peripheral blood, in HIV-infected patients (n = 30). **H**. Correlation between the frequency of HLA-DR^+^ CD38^+^ CD8^+^ T-cells that are CD107a^+^ after Gag peptides stimulation, the frequency of HLA-DR^+^ CD38^+^ CD8^+^ T-cells that are CD107a^+^ after PMA-ionomycin stimulation and the levels of plasma sCD14, in HIV-infected patients (n = 30). In C and D, The P value of the Dunn’s post-hoc test is shown. In E, G and H, the rho and P value of the Spearman test are shown.

To explore the factors associated with the altered cytotoxic program observed in CD8^+^ T-cells from HIV-infected patients in comparison with seronegative individuals, we performed a Principal Component Analysis (PCA) including the following evaluated parameters: phenotypic and functional characterization of CD8^+^ T-cells; length of the treatment; CD4:CD8 ratio, and plasma levels of the inflammation marker sCD14. When we evaluated the weight of variables of component 1 (which explains the 32.8% of the variability in the data), as expected, the frequencies of CD107a^+^ CD8^+^ T-cells, alone or co-expressed with IFN-γ or perforin, were variables with a high weight (Fig 6A). Interestingly, the length of treatment, in months, was also a variable of high component weight (Fig 6A). Indeed, when we divided HIV-infected patients in those with <24 or ≥25 months of treatment, the PCA yielded two separated clusters of patients, as well as a cluster of seronegative individuals (Fig 6B). Interestingly, after polyclonal stimulation, HIV-infected patients with ≥25 months of treatment had a significantly higher frequency of CD107a^+^ CD8^+^ T-cells than patients with <24 months therapy, reaching levels similar to those in seronegative individuals (Fig 6C). However, the effect of treatment duration was not evident in the case of granzyme B^+^ perforin^+^ CD8^+^ T-cells, as HIV-infected, irrespective of their treatment length, had higher frequencies of these cells compared with seronegative individuals (Fig 6D). Moreover, when we evaluated the effect of treatment duration on the response of HIV-specific CD8^+^ T-cells, a positive correlation was found between the frequency of CD107a^+^ HIV-specific CD8^+^ T-cells and the length of the treatment (Fig 6E). Accordingly, we observed a tendency for a higher in vitro cytotoxic capacity in HIV-specific CD8^+^ T-cells from patients with ≥25 months of treatment compared with those from patients with <24 months of therapy (Fig 6F).

Finally, similar results were obtained in HLA-DR/CD38-expressing subsets, although the effect of treatment duration was more evident in the HLA-DR-expressing subsets. Thus, compared with patients with <24 months of treatment, those with ≥25 months of therapy had higher frequencies of CD107a^+^ HLA-DR^+^ CD38^-^ and HLA-DR^+^ CD38^+^ cells (S8A and S8B Fig). In fact, in patients with ≥25 months of therapy, the frequency of the latter subset reached levels similar to those in seronegative individuals (S8A and S8B Fig). Non-significant differences were found for CD107a^+^ HLA-DR^-^ CD38^+^, and HLA-DR^-^ CD38^-^ cells between the HIV groups (S8C and S8D Fig). On the other hand, patients with ≥25 months of therapy had a tendency and a significant decrease, respectively, in the frequency of granzyme B^+^ perforin^+^ HLA-DR^+^ CD38^-^ and HLA-DR^+^ CD38^+^ cells, compared with patients with <24 months of therapy, without reaching levels similar to those in uninfected controls (S8E and S8F Fig). In contrast, patients with ≥25 months of therapy had increased frequencies of granzyme B^+^ perforin^+^ HLA-DR^-^ CD38^+^ and HLA-DR^-^ CD38^-^ cells (S8G and S8H Fig), suggesting a lack of effect of treatment duration in these HLA-DR^-^ subsets. Overall, these results indicate that the cytotoxic program of total and HIV-specific CD8^+^ T-cells from HIV-infected patients is modulated according to the duration of HAART, partially reaching levels similar to those in seronegative individuals. This effect is more evident in the degranulation capacity, and in HLA-DR-expressing subsets.

### The degranulation ability of CD8^+^ T-cells from HIV-infected patients is negatively associated with their exhaustion state, and with the systemic inflammation levels

Ultimately, we hypothesized that the exhaustion state of CD8^+^ T-cells (expression of the inhibitory receptor PD-1), and the systemic inflammation levels (measured by plasma sCD14) in HIV-infected patients were associated with the altered cytotoxic program of CD8^+^ T-cells in these individuals. Indeed, similar to previous reports [10,19], despite suppressive HAART, HIV-infected patients had higher frequencies of circulating total PD-1^+^ CD8^+^ T-cells, and plasma levels of sCD14 than seronegative individuals, without being influenced by treatment duration (S9A and S9B Fig). Interestingly, the frequency of HLA-DR^+^ CD38^+^ CD8^+^ T-cells that are CD107a^+^, but no other HLA-DR/CD38-expressing or total CD8^+^ T-cells, negatively correlated with the frequency of HLA-DR^+^ CD38^+^ CD8^+^ T-cells that are PD1^+^, evaluated in resting blood (Fig 6G). This association is supported by the highest expression of PD-1 in HLA-DR^+^ CD38^+^ CD8^+^ T-cells [26]. Indeed, compared with patients with <24 months of therapy, the frequency of HLA-DR^+^ CD38^+^ CD8^+^ T-cells that are PD-1^+^ in patients with ≥25 months of treatment, decreased to levels similar to those in seronegative individuals (S9C Fig), indicating that the major effect of the exhaustion state is on this particular subset, and it can improve with a long continuous treatment. Finally, the frequency of total and HIV-specific HLA-DR^+^ CD38^+^ CD8^+^ T-cells that are CD107a^+^, but no other HLA-DR/CD38-expressing or total CD8^+^ T-cells, were negatively correlated with the sCD14 levels (Fig 6H), further supporting a negative effect of the systemic inflammation on these subsets. In summary, the degranulation ability of CD8^+^ T-cells from HIV-infected patients, particularly those HLA-DR^+^ CD38^+^, is negatively modulated by their exhaustion state and with the systemic inflammation levels.

## Discussion

After activation, human circulating CD8^+^ T-cells rapidly up-regulate perforin, concomitant to degranulation and cytokine production [28]. Subsequently, the degranulation process causes the release of cytotoxic molecules at the immunological synapse–with the simultaneous decrease of intracellular granzyme B and perforin- contributing to the efficient induction of cell death [3]. However, our study evidences that the degranulation capacity of circulating CD8^+^ T-cells is decreased in HIV-infected patients, resulting in a lower release of cytotoxic granules and an increased intracellular expression of granzyme B and perforin. This functional defect remains despite HAART-induced viral suppression. In addition, similar to previous reports [15,18,48,49], HIV-infected patients on HAART exhibit persistently increased levels of circulating CD8^+^ T-cells, high immune exhaustion, chronic immune activation and systemic inflammation. Importantly, in this study, we used T-cell receptor (TCR)-independent (PMA-Ionomycin) and TCR-dependent antigen-specific (Gag peptides) stimulation to assess the cytotoxic potential of CD8^+^ T-cells from HIV-infected patients on suppressive HAART. CD8^+^ T-cell degranulation requires the activation of cellular Protein Kinases [50–52], which are activated by phorbol esters and calcium ionophores [52,53]. In addition, PMA-ionomycin stimulate de novo synthesis and granule accumulation of cytotoxic molecules in activated CD8^+^ T-cells [28], and trigger the lysis of nonantigen-bearing target cells [54,55]. Altogether, these data support the use of PMA-Ionomycin for the evaluation of the cytotoxic potential of CD8^+^ T-cells.

Similar to our results, some reports have demonstrated a higher expression of granzyme B and/or perforin in HIV-infected patients on HAART [36,37,56,57] or without treatment [16], compared with seronegative individuals. However, it has also been observed that perforin expression in HIV-, Epstein-Barr virus (EBV)- and Cytomegalovirus (CMV)-specific CD8^+^ T-cells is reduced in HIV-infected patients, and this defect is accompanied by a lower expression of granzyme B, and a less differentiated state in HIV-specific cells, but not in cells of other antigen specificities [20]. In addition, in untreated HIV-infected patients, as well as Simian Immune deficiency virus (SIV)-infected macaques, HIV/SIV-specific CD8^+^ T-cells exhibit a high cytotoxic potential (namely, high expression of perforin and granzyme B) in early infection, but this capacity decreases during chronic infection [58–60]. In contrast to our results, a previous report showed that CD8^+^ T-cells from treated HIV-infected patients have a lower expression of perforin and granzyme B after in vitro stimulation with interleukin (IL)-2 or the HIV Envelope protein compared with cells from untreated patients, suggesting that anti-retroviral drugs could have a negative effect in the synthesis of these cytotoxic molecules in CD8^+^ T-cells [61]. Overall, this would not be the case of total CD8^+^ T-cells from our cohort of patients under suppressive HAART, as an increased intracellular expression of cytotoxic molecules was evidenced. This could be related to the type of stimulation used in each study, as well as that most of our analyses were based on total, but not antigen-specific, CD8^+^ T-cells.

In accordance with our results, a low degranulation capacity of antigen-specific CD8^+^ T-cells in HIV-infected patients, in particular those exhibiting a progressive pattern, in whom a lower expression of CD107a after Gag peptide stimulation was observed, compared with long-term non-progressors patients [7]. HIV infection also impairs the degranulation capacity of *Mycobacterium tuberculosis*-specific CD8^+^ T-cells in individuals with untreated HIV and latent tuberculosis coinfection [62]. In addition, in Friend virus infected-mice, a model of chronic retroviral infection, CD8^+^ T-cells exhibit a degranulation dysfunction [63]. In contrast, other reports have demonstrated that CD8^+^ T-cells, from chronic untreated HIV-infected patients, maintain their degranulation capacity after superantigen and antigen-specific stimulation [64], whereas others have demonstrated a comparable degranulation capacity between CD8^+^ T-cells from different antigen specificities [45]. Importantly, although the surface expression of CD107a associates with the exocytosis of the cytotoxic molecules [2,35], it does not directly reflect the CD8^+^ T-cell cytotoxic capacity, as not all lytic granules contain perforin or granzyme B [4]. Indeed, a previous report showed that perforin can follow a granule-dependent or independent traffic to the immunological synapse, and it can be retained in early endosomal compartments after cell activation [28]. This could be the case for CD8^+^ T-cells in HIV-infected patients, which have been exposed to chronic activation and might accumulate a high content of perforin.

The cytotoxic capacity of human CD8^+^ T-cells varies according to their transcriptional profile and differentiation state. The Runt-related transcription factor 3 (Runx3), the T-box transcription factor (T-bet) and Eomesodermin (Eomes), in cooperation, regulate the expression of perforin, granzyme B and IFN-γ [65,66]. Indeed, the decrease in the cytotoxic potential of HIV and SIV-specific CD8^+^ T-cells during chronic infection is associated with the expansion of cells expressing low levels of T-bet [58,59]. Effector and effector memory cells have a high expression of the aforementioned transcription factors and a high cytotoxic potential, followed by central memory and naïve cells [4,5,65,67]. This is consistent with our data showing that most of CD8^+^ T-cells, responding to polyclonal and antigen-specific stimulation are HLA-DR^+^ cells, which are characterized by an effector memory profile, and, particularly, are composed of a high proportion of CD57^hi^ cells; in fact, this marker is highly associated with the expression of cytotoxic molecules [5,45,68]. However, our results suggest that an alteration in the cytotoxic program of CD8^+^ T-cells from HIV-infected patients mainly lies on effector memory HLA-DR^+^ cells, which could compromise the overall CD8^+^ T-cells response in these individuals, taking into account the increased frequencies of HLA-DR^+^ CD38^+/-^ CD8^+^ T-cells compared with seronegative controls [18,26,69]. Moreover, similar to previous reports [17,70], we observed a shift to a less differentiation profile in HLA-DR^+^ CD38^-/+^ (effector memory) CD8^+^ T-cells from HIV-infected patients, which could affect their functional capacity. This differentiation defect is particularly observed in HIV-specific cells (which are mainly composed of HLA-DR^+^ cells, according to our results and previous reports [33]) but not for EBV or CMV. Moreover, HIV-specific CD8^+^ T-cells are characterized by a low expression of perforin and high expression of CD27, a marker indicative of an early/intermediate differentiation state [20,70,71]. This defect does not seem to be restored by HAART [71,72]. Indeed, our results suggest a defect in the cytotoxic program of HIV-specific CD8^+^ T-cells despite HAART-induced viral suppression. Thus, the skewed differentiation profile of HIV-specific CD8^+^ T-cells confer them a lower cytotoxic potential [17,70].

In the context of HIV infection, the expression of an efficient cytotoxic program in circulating HIV-specific CD8^+^ T-cells is critical in the control of viral replication [7,8]. Of note, most of our results are based on polyclonally-stimulated CD8^+^ T-cells, and it could be possible that the cytotoxic program of circulating CD8^+^ T-cells from our cohort of HIV-infected patients in HAART is enough for disease control. Nonetheless, the association with the exhaustion and inflammatory state in HIV-infected patients, supports a cellular defect that could impair the CD8^+^ T-cells cytotoxic response, particularly in the setting of co-infections or malignancies. Interestingly, a previous study showed that the frequency of perforin^+^ HIV-specific CD8^+^ T-cells negatively correlates with the CD4^+^ T-cells count in treated and untreated patients, and the authors attributed this finding to the chronic activation in these individuals [73]. Indeed, CD8^+^ T-cells need to be in an activated/effector state for expression of cytotoxic molecules [74]. In addition, the high expression of perforin and/or granzyme B in CD8^+^ T-cells has been associated with disease severity/activity in patients with herpesvirus reactivation after organ transplantation, and in patients with systemic lupus erythematosus [75,76]; in the autoimmune condition, this cytotoxic profile was driven by cell activation [76]. Furthermore, we observed an increase in the degranulation capacity and the in vitro cytotoxicity of total and HIV-specific cells, along treatment time, but not of the expression of cytotoxic molecules, suggesting that reconstitution of these parameters by HAART is incomplete or needs a long treatment duration, as we previously reported [26]. Finally, is important to point that changes induced by HAART in the cytotoxic response of circulating CD8^+^ T-cells, by evaluating HIV-infected patients before and after treatment initiation, as well as the changes throughout therapy or after treatment failure, required further studies. In addition, it is necessary to clarify it the reported defects in CD8^+^ T-cells is restricted to HIV-specific cells or includes cells with different specificities.

## Conclusions

Despite the HAART-induced suppression of viral replication, circulating CD8^+^ T-cells from HIV-infected patients exhibit a defect in their degranulation capacity, with the subsequent lower extracellular release of cytotoxic molecules and their intracellular accumulation. Most of the alterations in the cytotoxic program of CD8^+^ T-cells from HIV-infected patients occur in total and HIV-specific cells expressing the activation marker HLA-DR, which have an effector memory profile and are the most prevalent subset. The functional defect of CD8^+^ T-cell subsets, particularly those expressing HLA-DR, was associated with the immune exhaustion and inflammatory state in HIV-infected patients, and only partially recovered with time after treatment, supporting an incomplete immune reconstitution with HAART and highlighting the need for other immunomodulatory interventions.

## Supporting information

S1 Fig **A.** Gating strategy for the analysis of activated CD8^+^ T-cells. Pseudo-color plots of a representative seronegative individual are shown. **B-E**. Frequencies of HLA-DR and CD38 (**B**), PD-1 (**C**), Annexin V (**D**), and perforin and/or granzyme B (**E**) CD8^+^ T-cells from seronegative individuals (n = 3) after culture without stimulation.
(TIFF)

S2 Fig Kinetics of the expression of HLA-DR and CD38 (**A**), PD-1 (**B**), non-viable cells (amine reactive dye^+^ cells) (**C**), perforin and/or granzyme B (**D**), CD107a alone or together with granzyme B or perforin (**E**) and IFN-γ alone or together with granzyme B or CD107a (**F**) in CD8^+^ T-cells from HIV-infected patients (n = 3) after stimulation with PMA-Ionomycin (at 50 and 500 ng/mL, respectively). In B, the expression of PD-1 in total and HLA-DR/CD38-expressing CD8^+^ T-cells is shown. *P = 0.04 vs 0 hours; &P = 0.04 vs 2 hours; #P = 0.04 vs 6 hours; +P = 0.04 vs 12 hours.
(TIFF)

S3 Fig **A.** Frequency of granzyme B^+^ perforin^+^ CD8^+^ T-cells in unstimulated cells from seronegative (n = 15) and HIV-infected (n = 30) individuals, after 12 hours culture. **B.** Proportion of granzyme B/perforin-expressing CD8^+^ T-cells from seronegative (n = 15) and HIV-infected (n = 30) individuals after 12 hours of PMA-ionomycin stimulation. *P = 0.01; ****P<0.0001; Seronegative vs HIV-infected individuals. **C**. Frequency of CD8^+^ T-cells that are CD107a^+^ granzyme B^+^ or perforin^+^ in seronegative (green dots, n = 15) and HIV-infected (red dots, n = 30) individuals. NS: Not statistically significant.
(TIFF)

S4 Fig **A.** Proportions of naïve, central memory (CM), effector memory (EM), and EM which express CD45RA (TEMRA) cells among resting CD8^+^ T-cells from seronegative and HIV-infected individuals. **B.** Proportion of TEMRA, naïve, CM, and EM cells among HLA-DR/CD38-expressing CD8^+^ T-cells from seronegative and HIV-infected individuals. **C.** Proportion of HLA-DR/CD38-expressing cells among TEMRA, naïve, CM, and EM cells CD8^+^ T-cells from seronegative and HIV-infected individuals. In all the cases, n = 3 in both groups of individuals; P value of the Mann-Whitney test. NS: Not statistically significant.
(TIFF)

S5 Fig Expression of granzyme B and IFN-γ in TEMRA, naïve, CM, and EM cells CD8^+^ T-cells after PMA-Ionomycin stimulation. Representative dot plots from a seronegative individual are shown above. The summary of results in 3 seronegative and 3 HIV-infected individuals are shown below. P value of the Mann-Whitney test. NS: Not statistically significant.
(TIFF)

S6 Fig **A.** Expression of perforin and CD107a in CD57^hi^, CD57^lo^ and CD57^-^ CD8^+^ T-cells from a representative seronegative individual after PMA-Ionomycin stimulation. The summary of the results is shown in right panels (n = 5). The P value of the Dunn’s post-hoc test is shown. **B-C**. Frequency of HLA-DR/CD38-expressing CD8^+^ T-cells that are CD107a^+^ perforin^+^ (**B**) or granzyme B^+^ (**C**) in seronegative (n = 15) and HIV-infected (n = 30) individuals, after PMA-Ionomycin stimulation. The P value of the Mann-Whitney test is shown. **D**. Frequency of granzyme B^+^ perforin^+^ CD8^+^ T-cells in unstimulated or Gag peptides-stimulated cells from HIV-infected patients (n = 30). The P value of the Wilcoxon test is shown. NS: Not statistically significant.
(TIFF)

S7 Fig Expression of perforin and CD107a in CD57^hi^, CD57^lo^ and CD57^-^ cells among HLA-DR/CD38-expressing CD8+ T-cells from a representative HIV-infected individual after Gag peptides stimulation, from a total of 5 individuals. (TIFF)

S8 Fig Frequencies of CD107a^+^ (**A-D**) and granzyme B^+^ perforin^+^ (**E-H**) HLA-DR^+^ CD38^-^ (**A** and **E**), HLA-DR^+^ CD38^+^ (**B** and **F**), HLA-DR^-^ CD38^+^ (**C** and **G**) and HLA-DR^-^ CD38^-^ (**D** and **H**) CD8^+^ T-cells in seronegative (n = 15) and HIV-infected individuals, the latter classified in those with <24 months or ≥25 months of therapy (n = 17 and n = 13, respectively). The P value of the Dunn’s post-hoc test is shown.
(TIFF)

S9 Fig **A.** Frequency of PD-1^+^ CD8^+^ T-cells in resting blood from seronegative and HIV-infected individuals. **B**. Levels of plasma sCD14 in seronegative and HIV-infected individuals. In A and B, the P value of the Mann-Whitney test is shown; n = 15 and n = 30 seronegative and HIV-infected individuals, respectively). **C**. Frequency of HLA-DR^+^ CD38^+^ CD8^+^ T-cells that are PD-1^+^ in resting blood from seronegative (n = 15) and HIV-infected individuals, the latter classified in those with <24 months or ≥25 months of therapy (n = 17 and n = 13, respectively). The P value of the Dunn’s post-hoc test is shown.
(TIFF)
