# Supplementary figures and images for: An altered cytotoxic program of CD8+ T-cells in HIV-infected patients despite HAART-induced viral suppression
Source: PLoS One. 2019 Jan 9;14(1):e0210540. doi: 10.1371/journal.pone.0210540 (PMC6326488; doi:10.1371/journal.pone.0210540)

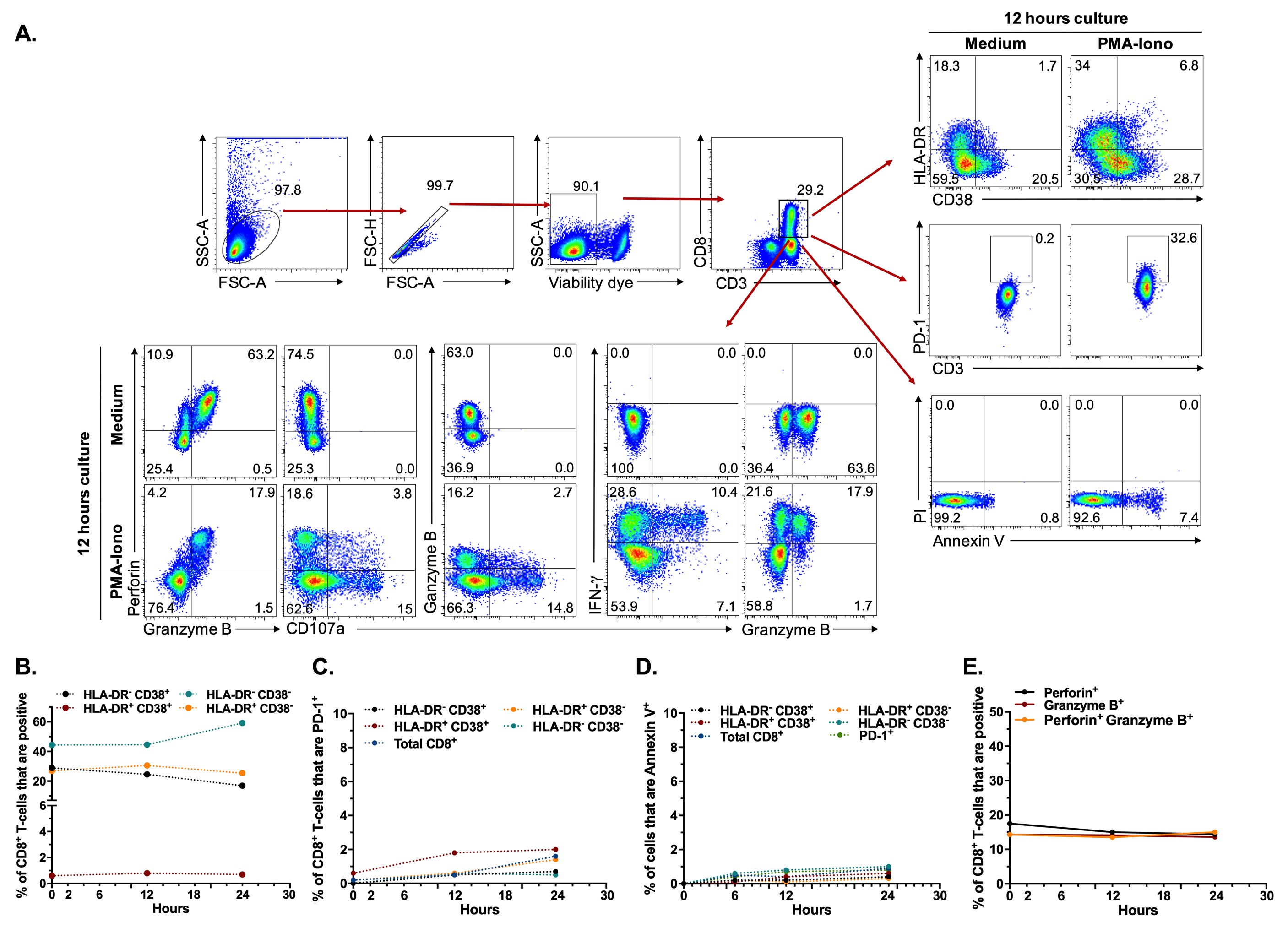

Supplement: S1 Fig — A. Gating strategy for the analysis of activated CD8+ T-cells. Pseudo-color plots of a representative seronegative individual are shown. B-E. Frequencies of HLA-DR and CD38 (B), PD-1 (C), Annexin V (D), and perforin and/or granzyme B (E) CD8+ T-cells from seronegative individuals (n = 3) after culture without stimulation. (TIFF) [file pone.0210540.s001.tiff]

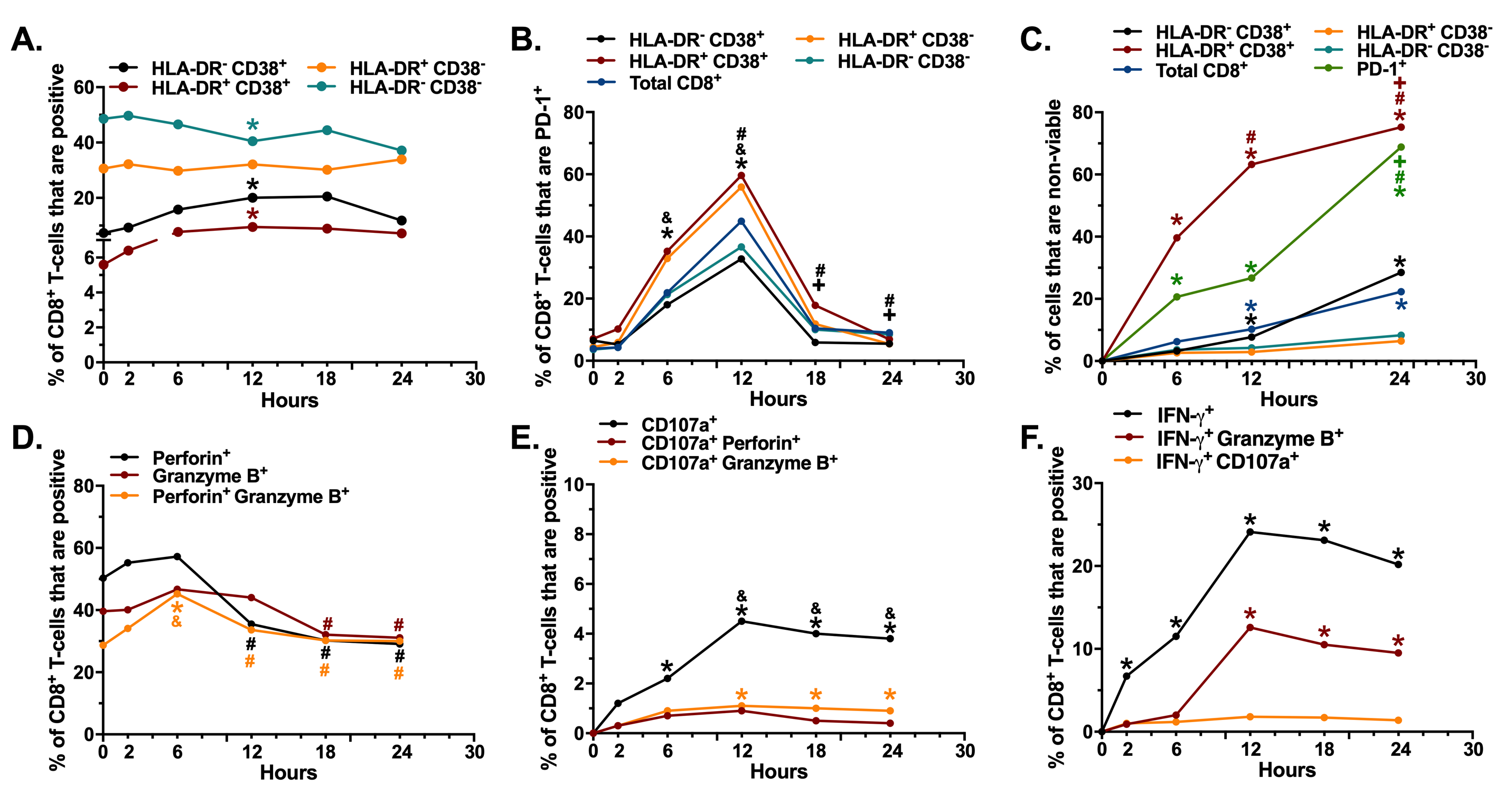

Supplement: S2 Fig — Kinetics of the expression of HLA-DR and CD38 (A), PD-1 (B), non-viable cells (amine reactive dye+ cells) (C), perforin and/or granzyme B (D), CD107a alone or together with granzyme B or perforin (E) and IFN-γ alone or together with granzyme B or CD107a (F) in CD8+ T-cells from HIV-infected patients (n = 3) after stimulation with PMA-Ionomycin (at 50 and 500 ng/mL, respectively). In B, the expression of PD-1 in total and HLA-DR/CD38-expressing CD8+ T-cells is shown. *P = 0.04 vs 0 hours; &P = 0.04 vs 2 hours; #P = 0.04 vs 6 hours; +P = 0.04 vs 12 hours. (TIFF) [file pone.0210540.s002.tiff]

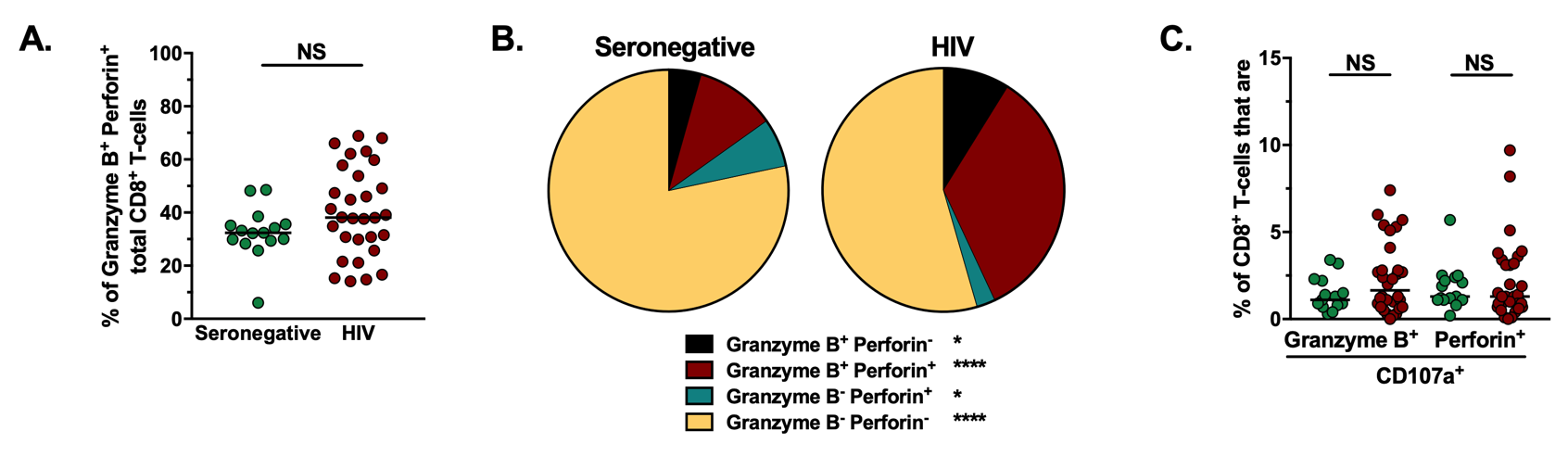

Supplement: S3 Fig — A. Frequency of granzyme B+ perforin+ CD8+ T-cells in unstimulated cells from seronegative (n = 15) and HIV-infected (n = 30) individuals, after 12 hours culture. B. Proportion of granzyme B/perforin-expressing CD8+ T-cells from seronegative (n = 15) and HIV-infected (n = 30) individuals after 12 hours of PMA-ionomycin stimulation. *P = 0.01; ****P<0.0001; Seronegative vs HIV-infected individuals. C. Frequency of CD8+ T-cells that are CD107a+ granzyme B+ or perforin+ in seronegative (green dots, n = 15) and HIV-infected (red dots, n = 30) individuals. NS: Not statistically significant. (TIFF) [file pone.0210540.s003.tiff]

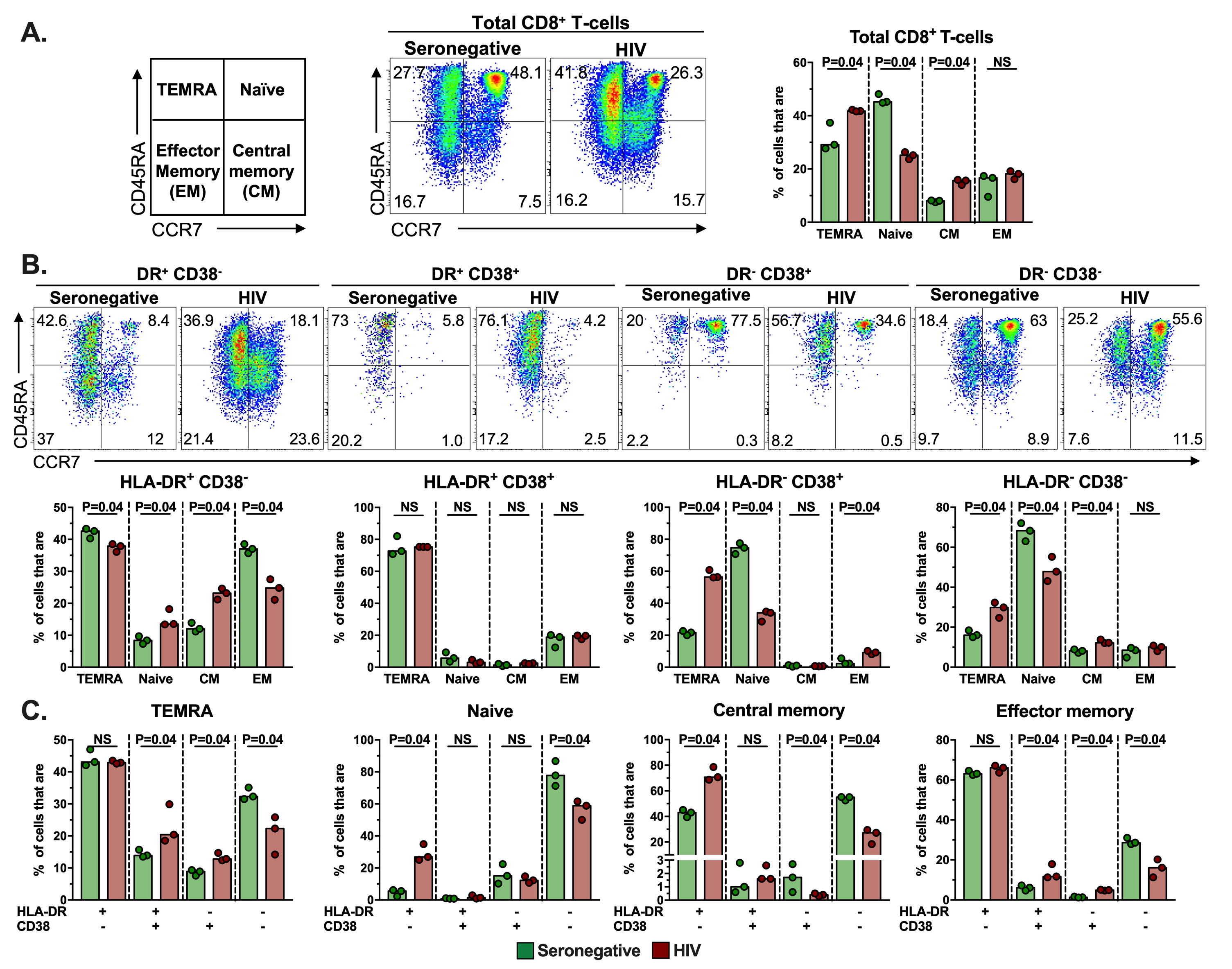

Supplement: S4 Fig — A. Proportions of naïve, central memory (CM), effector memory (EM), and EM which express CD45RA (TEMRA) cells among resting CD8+ T-cells from seronegative and HIV-infected individuals. B. Proportion of TEMRA, naïve, CM, and EM cells among HLA-DR/CD38-expressing CD8+ T-cells from seronegative and HIV-infected individuals. C. Proportion of HLA-DR/CD38-expressing cells among TEMRA, naïve, CM, and EM cells CD8+ T-cells from seronegative and HIV-infected individuals. In all the cases, n = 3 in both groups of individuals; P value of the Mann-Whitney test. NS: Not statistically significant. (TIFF) [file pone.0210540.s004.tiff]

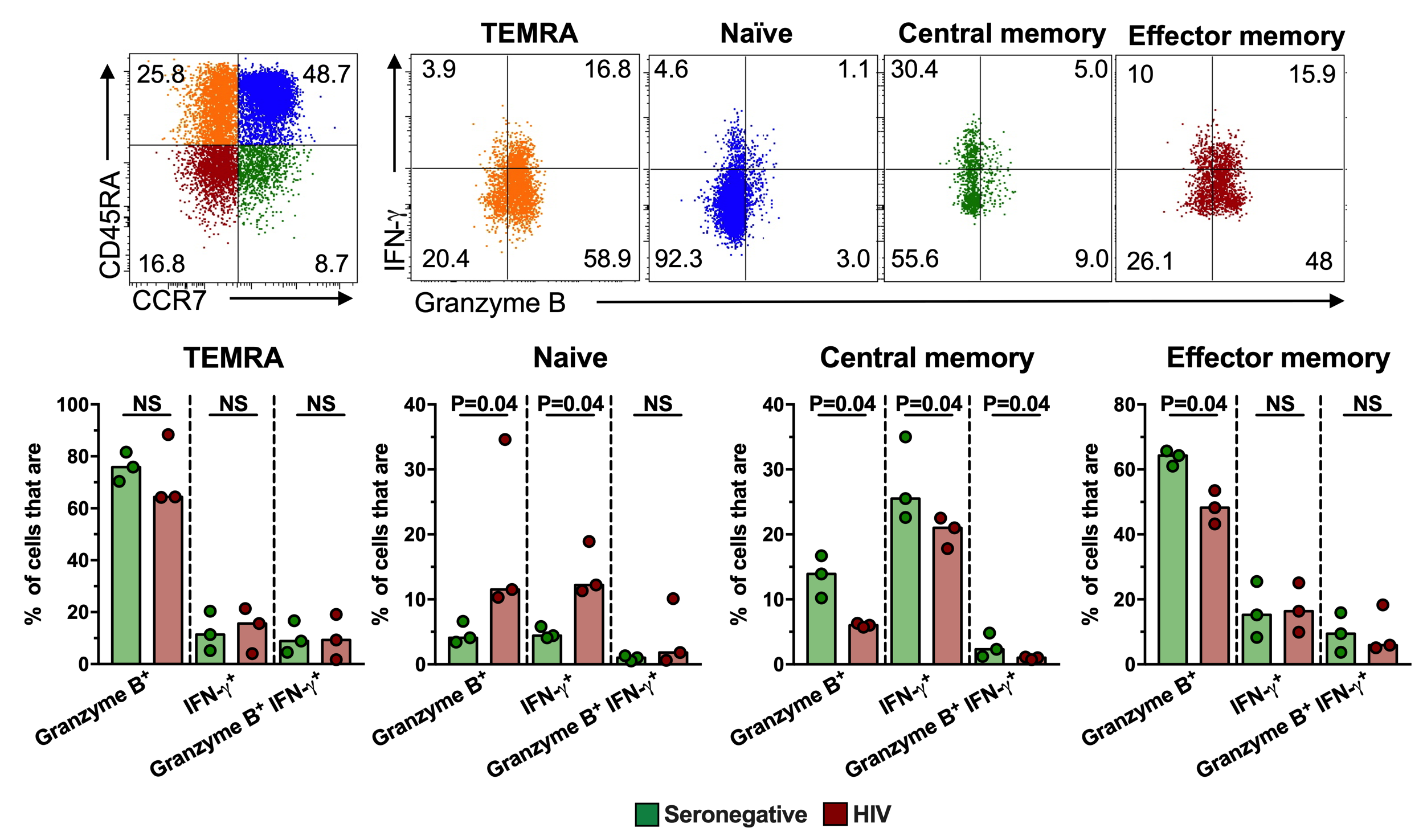

Supplement: S5 Fig — Representative dot plots from a seronegative individual are shown above. The summary of results in 3 seronegative and 3 HIV-infected individuals are shown below. P value of the Mann-Whitney test. NS: Not statistically significant. (TIFF) [file pone.0210540.s005.tiff]

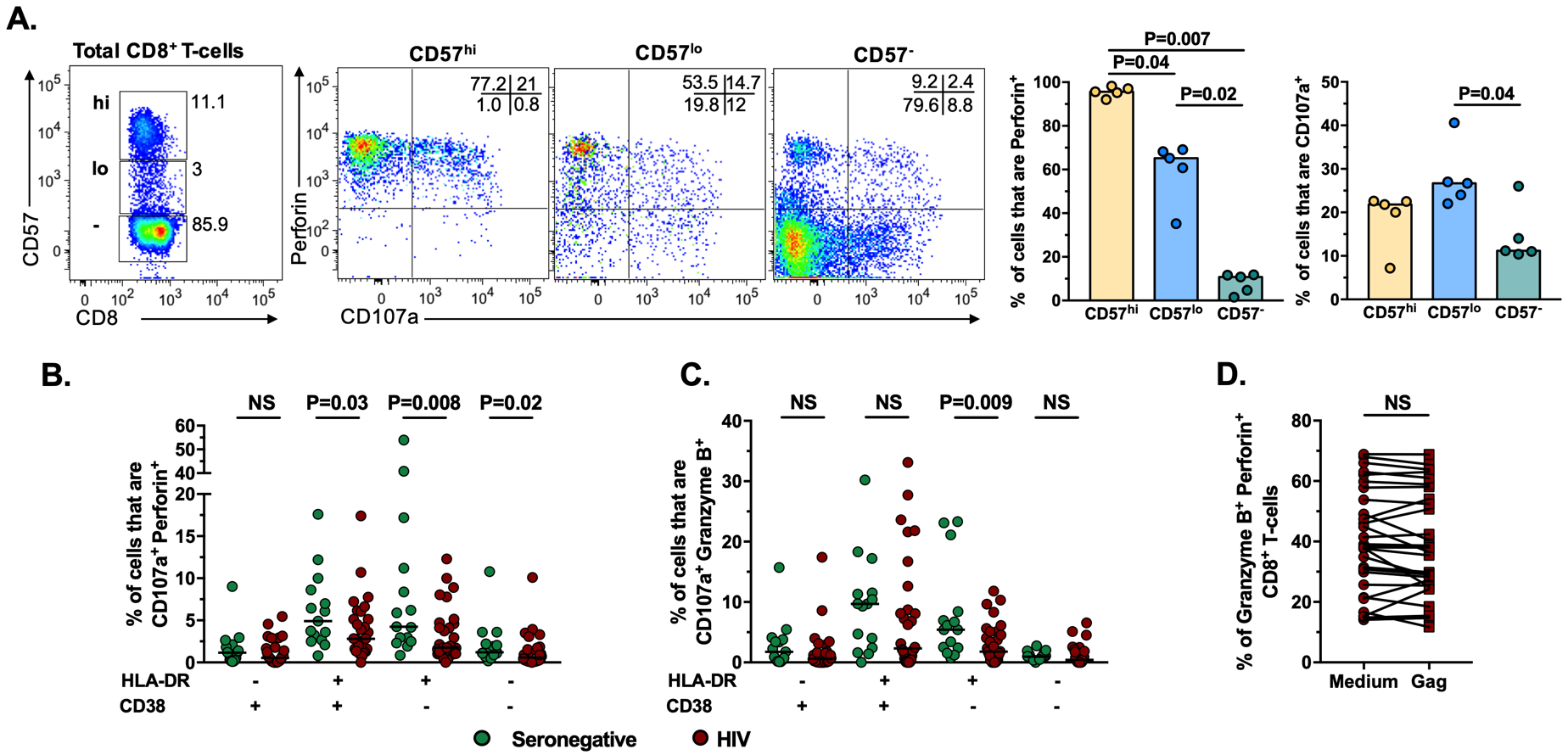

Supplement: S6 Fig — A. Expression of perforin and CD107a in CD57hi, CD57lo and CD57- CD8+ T-cells from a representative seronegative individual after PMA-Ionomycin stimulation. The summary of the results is shown in right panels (n = 5). The P value of the Dunn’s post-hoc test is shown. B-C. Frequency of HLA-DR/CD38-expressing CD8+ T-cells that are CD107a+ perforin+ (B) or granzyme B+ (C) in seronegative (n = 15) and HIV-infected (n = 30) individuals, after PMA-Ionomycin stimulation. The P value of the Mann-Whitney test is shown. D. Frequency of granzyme B+ perforin+ CD8+ T-cells in unstimulated or Gag peptides-stimulated cells from HIV-infected patients (n = 30). The P value of the Wilcoxon test is shown. NS: Not statistically significant. (TIFF) [file pone.0210540.s006.tiff]

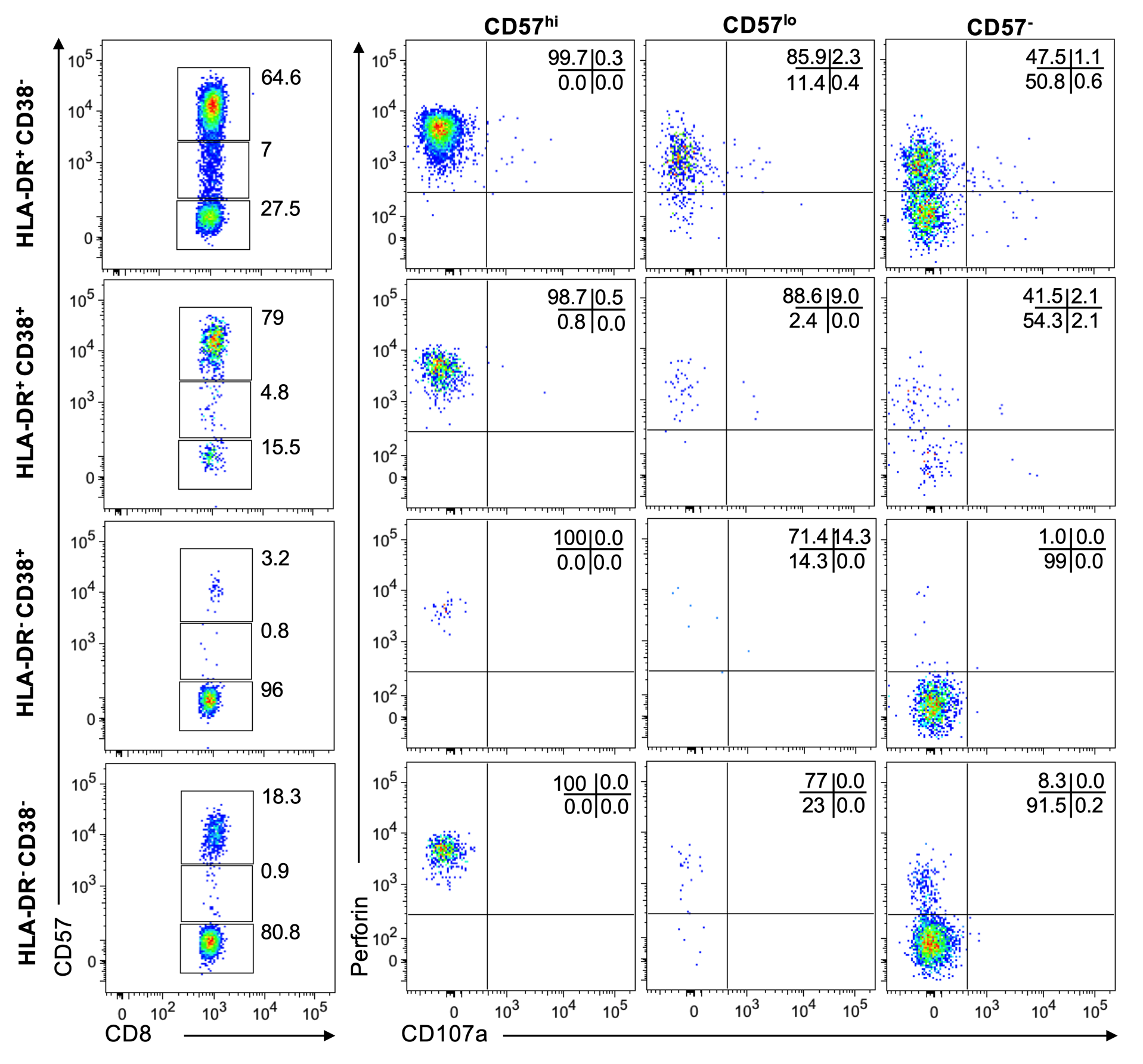

Supplement: S7 Fig — (TIFF) [file pone.0210540.s007.tiff]

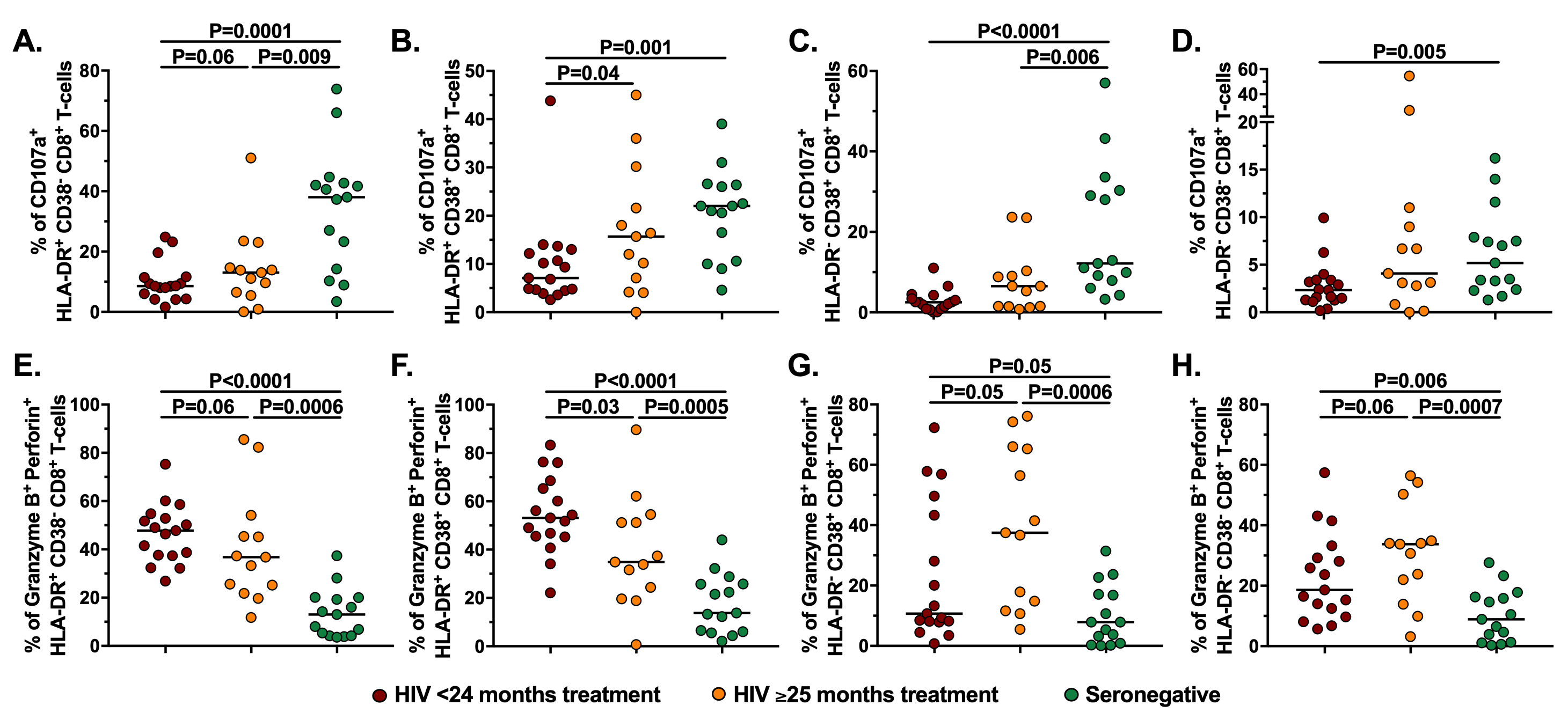

Supplement: S8 Fig — Frequencies of CD107a+ (A-D) and granzyme B+ perforin+ (E-H) HLA-DR+ CD38- (A and E), HLA-DR+ CD38+ (B and F), HLA-DR- CD38+ (C and G) and HLA-DR- CD38- (D and H) CD8+ T-cells in seronegative (n = 15) and HIV-infected individuals, the latter classified in those with <24 months or ≥25 months of therapy (n = 17 and n = 13, respectively). The P value of the Dunn’s post-hoc test is shown. (TIFF) [file pone.0210540.s008.tiff]

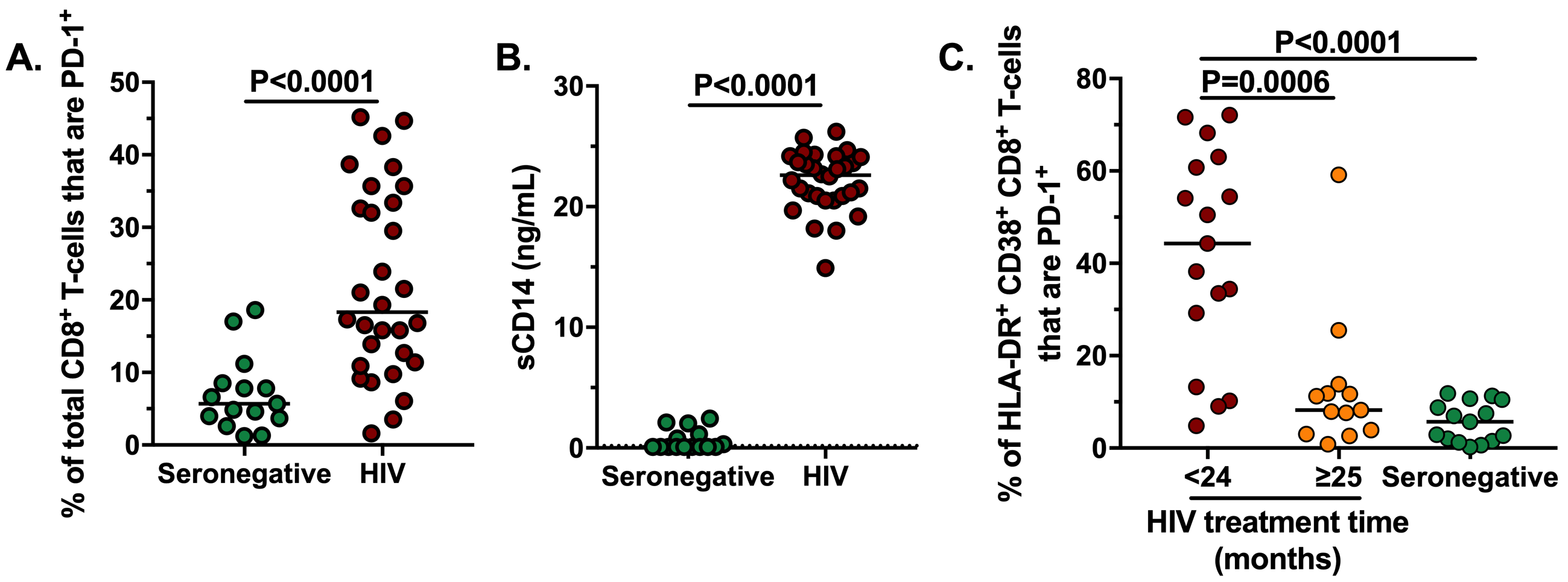

Supplement: S9 Fig — A. Frequency of PD-1+ CD8+ T-cells in resting blood from seronegative and HIV-infected individuals. B. Levels of plasma sCD14 in seronegative and HIV-infected individuals. In A and B, the P value of the Mann-Whitney test is shown; n = 15 and n = 30 seronegative and HIV-infected individuals, respectively). C. Frequency of HLA-DR+ CD38+ CD8+ T-cells that are PD-1+ in resting blood from seronegative (n = 15) and HIV-infected individuals, the latter classified in those with <24 months or ≥25 months of therapy (n = 17 and n = 13, respectively). The P value of the Dunn’s post-hoc test is shown. (TIFF) [file pone.0210540.s009.tiff]
